# Pyrrolopyrimidines: Design, Synthesis and Antitumor Properties of Novel Tricyclic Pyrrolo [2,3-*d*]pyrimidine Derivatives

**DOI:** 10.3390/molecules30142917

**Published:** 2025-07-10

**Authors:** Buer Song, Zarifa Murtazaeva, Lifei Nie, Rustamkhon Kuryazov, Shukhrat Gaybullaev, Chao Niu, Khurshed Bozorov, Haji Akber Aisa, Jiangyu Zhao

**Affiliations:** 1 State Key Laboratory Basis of Xinjiang Indigenous Medicinal Plants Resource Utilization, Xinjiang Technical Institute of Physics and Chemistry, Chinese Academy of Sciences, South Beijing Rd 40-1, Urumqi 830011, China; 2 Department of Organic Synthesis and Bioorganic Chemistry, Institute of Biochemistry, Samarkand State University, University Blvd. 15, Samarkand 140104, Uzbekistan; 3 Department of Chemistry, Urgench State University, Kh. Olimjon st. 14, Urgench 220100, Uzbekistan

**Keywords:** antitumor activity, carbonyl-amine condensation, DDR2 receptor, HT-29 cell line, molecular docking, *N*-halosuccinimide, pyrrolo[2,3-*d*]pyrimidine, pyrrolo[2,3-*d*]pyrimidine-imines, Tf_2_O, 7-deazapurine

## Abstract

The pyrrolo[2,3-*d*]pyrimidine (7-deazapurine) scaffold is a unique heterocyclic system included in the composition of most nucleotides. In this study, series of the pyrrolo[2,3-*d*]pyrimidine-imines and 3-*halo*-substituted pyrrolo[2,3-*d*]pyrimidines were designed and prepared in high yields. Condensed pyrimidines are obtained via carbonyl-amine condensation and carbon-halogen bond formation. Pyrrolo[2,3-*d*]pyrimidine-imines containing a bromine substituent at position C-4 of the phenyl ring and azepine side-ring exhibited superior antitumor activity on the colon cancer HT-29 cell line; IC_50_ values were 4.55 and 4.01 µM, respectively. These results revealed an interesting pattern, where condensed pyrimidinones containing an azepine ring demonstrated selective antitumor activity on the colon cancer cell line HT-29. In addition, the molecular docking results suggest that compound **8g** provided a thorough understanding of its interactions with the DDR2 active site. This could pave the way for further development and optimization of DDR-targeting drugs, contributing to advancements in cancer therapeutics. This lead compound may serve as design templates for further studies.

## 1. Introduction

**Figure 1:**
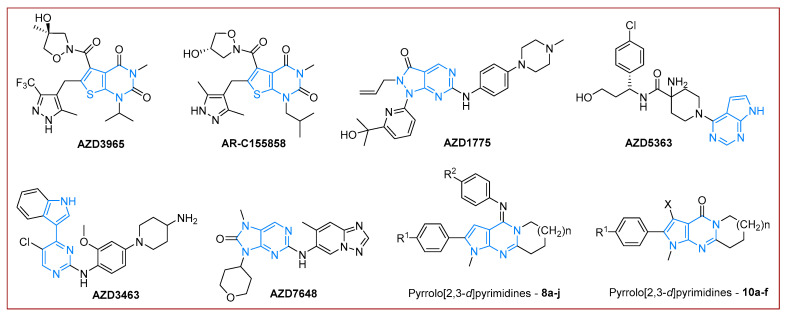
Structures of pyrimidine-based inhibitors from the AZD series used in cancer treatment, and the pyrrolo[2,3-*d*]pyrimidines **8a**–**j** and **10a**–**f** synthesized in this work.

Pyrimidine-based small molecules containing various five-membered heterocycles have been successfully used in clinical trials as antitumor inhibitors [1,2,3]. These include the monocarboxylate transporter 1 (MCT1) inhibitors AZD3965 and AR-C155858 [4,5,6,7], the WEE1 inhibitor AZD1775 [8,9], the anaplastic lymphoma kinase (ALK) inhibitors AZD3463 and AZD5363 [10,11,12,13], the DNA-dependent protein kinase (DNA-PK) inhibitor AZD7648 [14,15,16], and other AZD-related inhibitors [17,18] employed in cancer treatment (Figure 1).

The pyrrolo[2,3-*d*]pyrimidine (7-deazapurine) [19] scaffold is a unique heterocyclic system included in the composition of most nucleotides [20,21]. This includes DNA and RNA, plants [22], and biologically critical synthetic derivatives [23,24,25,26,27]. These condensed heterocyclic compounds contain a six-membered pyrimidine portion and a five-membered pyrrole ring. Tricyclic pyrrolo[2,3-*d*]pyrimidines have been shown to inhibit the BCL6 BTB domain protein-protein interaction [28]. We recently reported a facile synthesis of tricyclic pyrrolo[2,3-*d*]pyrimidinones containing 2-substituted-phenyl fragments in the pyrrole ring in high yield [29]. Antitumor evaluation of substituted 2-phenyl pyrrolo[2,3-*d*]pyrimidinones revealed enhanced antitumor activity due to the introduction of a phenyl group at position 2 of the pyrrole core and the presence of halogen substituents in the 2-phenyl portion. The importance of the 2-phenyl ring was confirmed during the investigation of the oxazolo[5,4-*d*]pyrimidine library [30].

**Figure 2:**
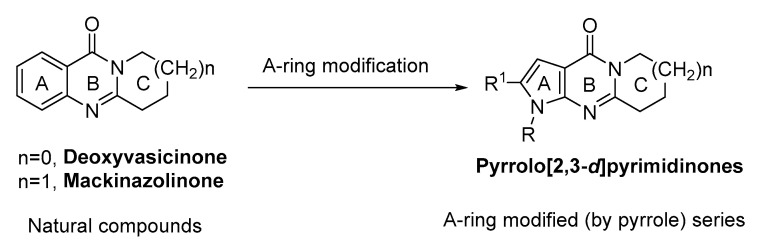
Natural-products-based derivatization.

Our research group has extensive experience and interest in *N*-heterocycles, especially in condensed pyrimidines [31,32,33,34,35,36,37,38,39,40]. We have studied their biological properties using various targets [41] and have probed their antitumor activity against human cancer cell lines [42]. The tricyclic pyrrolo[2,3-*d*]pyrimidine molecule contains several reaction centers; these include the nitrogen heteroatoms of the pyrimidine and pyrrole rings, the carbonyl group of the pyrimidine core, and the methylene side-ring. The presence of these substituents contributes to the increased reactivity of these electrophilic substitution reactions. These factors also contribute to the formation of deazapurine in the biosynthesis process and in the synthesis of various intermediates for the total synthesis of desired natural products and synthetic compounds. In addition, the tricyclic pyrrolo[2,3-*d*]pyrimidinones that are the subject of this research are synthetic analogues of the deoxyvasicinone [40,43] and mackinazolinone [44] alkaloids, where a pyrrole ring replaces the benzene ring (A-ring) (Figure 2).

We designed our tricyclic pyrrolo[2,3-*d*]pyrimidine-based compounds based on the presence of this core structure in several kinase-targeting scaffolds used in modern oncology treatments. In addition to the previously discussed structures, our design incorporates essential pharmacophoric components commonly found in FDA-approved anticancer drugs [45,46]. These components include a fused *N*-heterocycle that mimics purines or quinazolines (e.g., gefitinib and imatinib) [47,48]; electron-rich aryl substitutions that facilitate π-stacking interactions; and halogen groups that enhance binding affinity and metabolic stability. This combination reflects the rational hybridization of privileged fragments, aiming to retain drug-likeness while exploring novel spatial geometry and electronic distribution for selective anticancer activity.

Thus, we proceeded here to examine the chemical modification of the pyrrolo[2,3-*d*]pyrimidine core with a tricyclic ring, where we synthesized its imines via carbonyl-amine condensation and studied carbon-halogen bond formation at position C-3 of the pyrrole ring. All synthesized novel compounds were tested for their antitumor activity on human cancer cell lines, including HeLa (cervical), MCF-7 (breast), and HT-29 (colon).

## 2. Results and Discussion

### 2.1. Synthesis

**Scheme 1:**
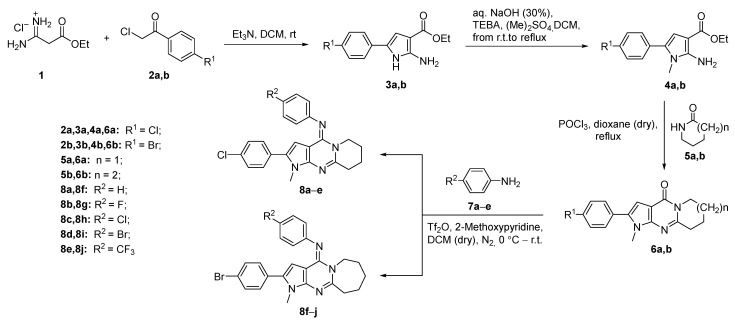
Synthesis of pyrrolo[2,3-*d*]pyrimidine-imines **8a**–**j**.

*ipso*-Imination of ketone, carbonyl, and sulfoxide functional groups with amines (1°–3°) allows rapid access to the synthesis of imine-tethered compounds [49,50,51]. Tricyclic pyrrolo[2,3-*d*]pyrimidinones **6a** and **6b** contain the C=O group. Together with the neighboring nitrogen heteroatom of the pyrimidine ring, this moiety becomes an amide-type functional fragment. Therefore, we studied the *ipso*-imination of these tricyclic pyrimidinone ring systems via carbonyl-amine condensation. The three-step total synthesis of **6a** and **6b** was described in our earlier report. Trifluoromethanesulfonic anhydride (Tf_2_O) [52] was selected as an amide activation agent for carbonyl-amine condensation. Tf_2_O is an effective electrophilic activation agent for the cyclic amides [53], the chemo-selective synthesis process of imines [54], and even in nucleophilic substitution reactions towards various generated phosphates [55]. Except for an aniline, 4-*halo*-substituted phenyl anilines (**7a**–**e**) were selected for the condensation. The base additive 2-methoxypyridine was also used. The carbonyl-amine condensation was performed using dichloromethane (DCM) as a reaction solvent at low temperatures, from 0 °C to r.t. (25 °C). The transformation easily occurred under the above conditions, and the desired pyrrolo[2,3-*d*]pyrimidine-imines **8a**–**j** were formed in good yield (45–99%) (Scheme 1). In summary, Tf_2_O/2-methoxypyridine-mediated carbonyl-amine condensation provided a convenient and mild pathway to pyrrolo[2,3-*d*]pyrimidine-imines **8a**–**j** formation. No other condition for *ipso*-imination was investigated in these systems.

It should be noted that the number of side-methylene rings of the pyrimidine system is not impacted for reactions yielding imines.

**Scheme 2:**
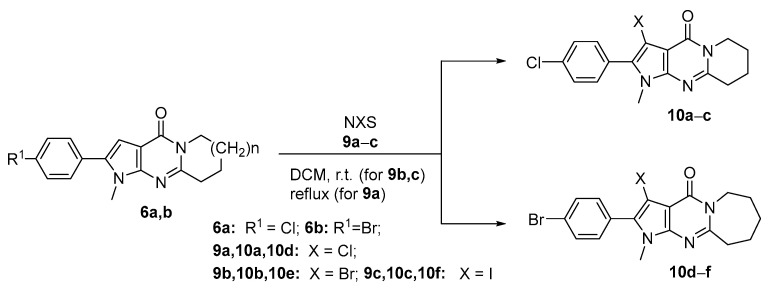
Synthesis of 3-halo-substituted pyrrolopyrimidinones **10a**–**f**.

The introduction of novel carbon-carbon bonds in heterocyclic systems can be implemented using various Pd-catalyzed reactions in the presence of halo-tethered intermediates. In this context, we performed carbon-halogen bond formation in the tricyclic pyrrolo[2,3-*d*]pyrimidine system. Three types of *N*-halosuccinimides were used in electrophilic aromatic halogenations resulting in carbon-halogen bond formation. These are *N*-chlorosuccinimide (NCS) (**9a**), *N*-bromosuccinimide (NBS) (**9b**), and *N*-iodosuccinimide (NIS) (**9c**), all of which are common sources of halonium ions due to their ease of handling and low toxicities [56]. Thus, if pyrrolopyrimidinones (**6a** and **6b**) and *N*-halosuccinimides (**9a**–**c**) were mixed in DCM, after stirring at room temperature for 1 h (**9b,c**) or reflux for 1 h (**9a**), the desired 3-halo-substituted pyrrolopyrimidinones **10a**–**f** were produced (Scheme 2) in yields up to 99%.

**Figure 3:**
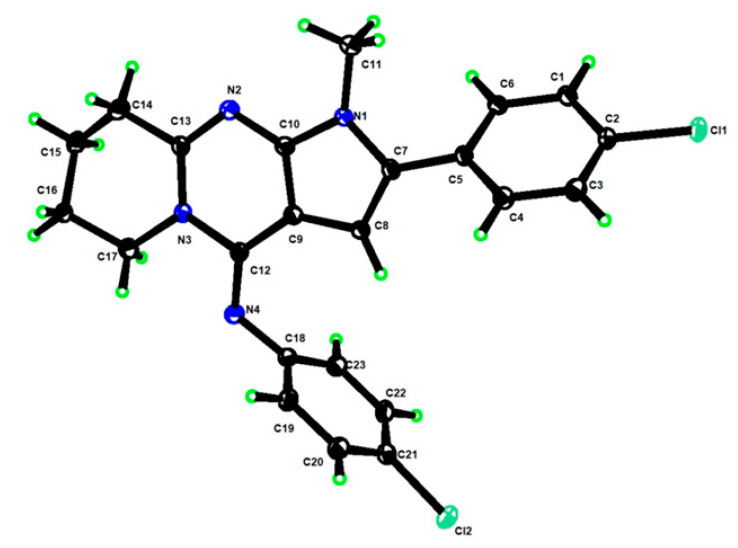
Crystal structure of compound **8c** (CCDC: 2169282).

All synthesized pyrrolo[2,3-*d*]pyrimidine-imines **8a**–**j** and **10a**–**f** were elucidated by ^1^H-NMR, ^13^C-NMR, and HRMS as described in the experimental section (see Supplementary Information), as well as X-ray analysis for (*E*)-*N*,2-*Bis*(4-chlorophenyl)-1-methyl-6,7,8,9-tetrahydropyrido[1,2-*a*]pyrrolo[2,3-*d*]pyrimidin-4(1*H*)-imine (**8c**) (Figure 3).

In the ^1^H NMR spectra of the target compounds **8a**–**8j**, the formation of the imine moiety was confirmed by the appearance of a characteristic singlet at δ 5.06–5.27 ppm, corresponding to the vinylic proton (=CH−), which is absent in the precursor compounds **6a** and **6b**. This signal is slightly deshielded due to conjugation with both aromatic and imine systems. The *N*-methyl protons appear consistently as a singlet at δ 3.59–3.70 ppm in all imine-containing compounds. Aliphatic methylene protons of the saturated rings (CH_2_-CH_2_-CH_2_) exhibit multiplet signals in the δ 1.90–4.20 ppm range, showing minimal but noticeable chemical shift differences compared to the precursors. Notably, the β-CH_2_ group (adjacent to the imine moiety) shows a downfield shift by approximately 0.10–0.15 ppm, likely due to the electron-withdrawing effect of the imine linkage. The aromatic regions (δ 6.80–7.60 ppm) of **8a**–**8j** reflect the presence of two substituted aryl rings: the fixed 2-(4-halophenyl) group and the variable *N*-aryl or *N*-heteroaryl substituent. Fluorinated (**8b**, **8g**) and trifluoromethyl (**8e**, **8j**) derivatives display additional fine splitting caused by ^19^F-^1^H couplings, along with downfield shifts in both aromatic and aliphatic protons, indicating increased deshielding. In compounds **10a**–**10c** (containing Cl, Br, I at position 3), the β-CH_2_ protons are observed around δ 2.48–2.50 ppm, while the CH_2_-N moiety appears as a triplet near δ 3.70 ppm. The *N*-methyl signal remains as a singlet at δ 3.10 ppm, shifted upfield compared to the imine-containing compounds. These changes confirm the substitution pattern on the pyrimidinone ring and suggest the absence of conjugated double bonds. The ^13^C NMR spectra of **8a**–**8j** show the imine carbon (C=N) resonating from δ 151–154 ppm, while other quaternary sp^2^ carbons (C2, C4) appear at δ 145–149 ppm. The *N*-methyl carbon consistently shows up at δ 43.8–44.4 ppm. Aliphatic CH_2_ carbons show signals from δ 19.3–32.5 ppm, with slight deshielding in compounds bearing bulky or electron-withdrawing aryl groups at the imine nitrogen. In the CF_3_-substituted compounds (**8e**, **8j**), additional signals for quaternary carbons and trifluoromethyl carbons are found in the δ 123–126 ppm range (q, J = 3.5–3.8 Hz), consistent with characteristic C-F couplings. The halo-containing compounds display a distinctive pattern: the carbonyl carbon (C=O) appears at δ 157–159 ppm, replacing the C=N signal. Adding halogens at position 3 results in new signals in the δ 90–106 ppm range, consistent with halogenated quaternary carbons. Notably, the iodine-bearing carbon in **10c** and **10f** shows the most downfield shift (δ ~ 106.05 ppm), aligning with literature data for iodinated heterocycles. HRMS confirmed the molecular formulas of all synthesized compounds, with the observed [M + H]^+^ signals closely matching the calculated values (within ≤3 mDa), confirming the proposed structures.

### 2.2. Antitumor Activity and Structure-Activity Relationship (SAR)

All the synthesized tricyclic pyrrolo[2,3-*d*]pyrimidines **8a**–**j** and **10a**–**f** were evaluated for their cytotoxic activity on cancer cell lines derived from the most common cancers: breast (MCF-7), cervical (HeLa), and colon (HT-29). Human cancer cell lines were treated with synthesized compounds, using doxorubicin (DOX) as a positive control, at concentrations of the compounds ranging from 0.001 to 6.561 μM. Cells were incubated at 37 °C for 72 h before measurement, and IC_50_ values were determined graphically. In our previous research [29], the cytotoxic activity of compounds **6a** and **6b** was also evaluated on the same human cancer cell lines used in the current study. Compound **6a** exhibited an IC_50_ value of 6.55 ± 0.31 µM, while the IC_50_ for compound **6g** was 7.61 ± 0.31 µM, against HeLa and HT-29 cell lines, respectively [29]. Table 1 shows that, among all tested pyrrolo[2,3-*d*]pyrimidines, only halo-compounds **10a** and **10b** displayed moderate activity against HeLa and MCF-7 cell lines, respectively. Other compounds were inactive and showed IC_50_ values ≥50 µM against these two cell lines. Among the tetrahydro derivatives, only compound **8a** (R_2_ = H) exhibited moderate inhibition (IC_50_ = 19.22 µM), indicating that substitution at the *N*-aryl group is crucial. The introduction of electron-withdrawing groups (F, Cl, Br, and CF_3_) at this position (as seen in **8b**–**8e**) resulted in a loss of activity. However, among the sixteen pyrrolo[2,3-*d*]pyrimidines synthesized, seven derivatives showed weak to high antitumor potency against HT-29 cell lines with IC_50_ values ≤50. Pyrrolo[2,3-*d*]pyrimidine-imines containing a bromine substituent at position C-4 of the phenyl ring and pentamethylene side-ring, (*E*)-2-(4-Bromophenyl)-1-methyl-*N*-phenyl-1,6,7,8,9,10-hexahydro-4*H*-pyrrolo[2′,3′:4,5]pyrimido-[1,2-*a*]azepin-4-imine (**8f**) and (*E*)-2-(4-Bromophenyl)-*N*-(4-fluorophenyl)-1-methyl-1,6,7,8,9,10-hexahydro-4*H*-pyrrolo[2′,3′:4,5]pyrimido[1,2-*a*]azepin-4-imine (**8g**), exhibited significant antitumor activity on HT-29 cell lines, with IC_50_ values 4.55 ± 0.23 and 4.01 ± 0.20 µM, respectively (Table 1).

It should be noted that both hexahydro derivatives have a bromine atom at R_1_ and either H or F at R_2_. This highlights the favorable impact of ring saturation and halogenation. However, further substitution at R_2_ diminished activity in **8h**–**8j**. Among the halo-containing analogues, compound **10a** exhibited modest activity across all three cell lines (IC_50_~23–28 µM), whereas **10b**, **10c**, **10e**, and **10f** displayed selective yet weaker activity. The presence of halogens (Br, I) at position 3 in the pyrrole ring appeared to be beneficial for cytotoxicity. Overall, SAR trends suggest that the cytotoxic potential is enhanced by a hexahydro scaffold, minimal substitution on the *N*-aryl ring, and selective halogenation, particularly for targeting colon cancer cells.

Derivatives **8f** and **8g** exhibited selective cytotoxicity against HT-29 colon cancer cells, with respective IC_50_ values of 4.55 ± 0.23 µM and 4.01 ± 0.20 µM. On the other hand, the inactivity of these leads on HeLa and MCF-7 cells (IC50 ≥ 50 µM) indicates specific tumor type selectivity. To assess their selectivity index (SI), the lead compounds were tested on non-cancerous human embryonic kidney (HEK-293) cells, yielding IC_50_ values of 195 ± 0.07 µM and 169 ± 0.10 µM for derivatives **8f** and **8g**, respectively. The selectivity index (SI) was then calculated as the ratio of the IC_50_ values for HEK-293 and HT-29 cells and is shown in Table 2.

**Figure 4:**
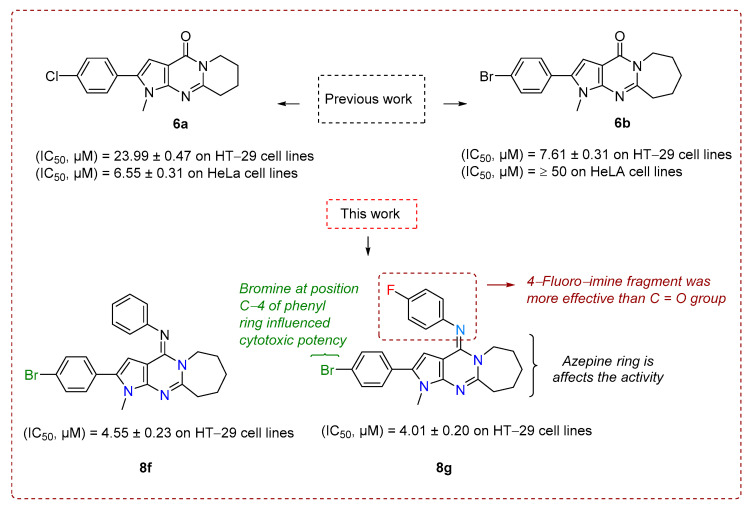
The SAR of the highly active compounds **8f** and **8g**.

When comparing the previous [29] and the present work, an interesting and consistent pattern emerges, indicating that the synthesized pyrrolo[2,3-*d*]pyrimidines exhibit a selective affinity for colon HT-29 cells. This highlights a crucial factor in the development of anticancer drugs. In addition, the bromine substituent at position C-4 of the phenyl ring enhanced cytotoxic potency, while the 4-fluoro-imine fragment was more effective than the carbonyl (C=O) group (Figure 4). An increase in the number of methylene (-CH_2_-) groups results in more selective antitumor compounds; here, the azepine ring affects the activity. With SI values exceeding 42, both compounds demonstrate favorable selectivity toward cancer cells over normal cells. Although these compounds have lower overall antiproliferative potency than doxorubicin (IC_50_: 0.18–0.94 µM), their significantly higher selectivity indices suggest an improved therapeutic window, particularly for colon cancer models, for **8f** and **8g**. The mechanism of action of these potential antitumor compounds is being investigated to elucidate their further antitumor properties.

### 2.3. Docking Study

**Figure 5:**
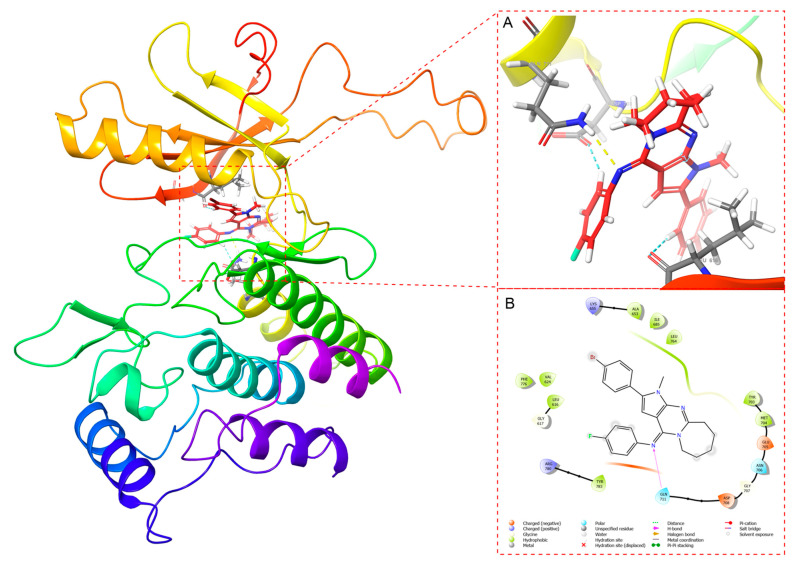
Docking result (**A**) and the 2D diagram (**B**) of **8g** with DDR2.

Discoidin domain receptors (DDRs) respond to collagen and play a crucial role in many cellular processes [57,58]. These activities include shaping tissues, promoting cell growth and differentiation, facilitating cell adhesion, and enabling tissue movement and repair [2]. Note that DDRs are a type of receptor tyrosine kinase (RTK) belonging to the same enzyme family as the epidermal growth factor receptor (EGFR). Although compounds **8f** and **8g** exhibited similar biological activities, **8g** was chosen for docking due to its fluorine atom, which influences molecular recognition and binding interactions in silico. Compound **8g** and avitinib (an EGFR receptor) have a similar structure; avitinib contains a pyrrolopyrimidine system. Several *N*-heterocycles, including pyrimidine [59], pyrazole [2,60,61], and pyrrolopyrimidines, have been shown to inhibit DDR1 or DDR2 [62]. Molecular docking studies using the Schrödinger platform [63,64] have identified compound **8g** as a promising anticancer agent focused on the DDR2 active site. The docking simulations used the OPLS4 force field and the DDR2 X-ray crystal structure, which was obtained from the Protein Data Bank (PDB entry 6FER) [65]. The method was validated using a redocking procedure with the co-crystallized ligand. The docking analysis of compound **8g** yielded a score of -7.856 kcal/mol, indicating its strong binding potential through multiple key interactions. Notable hydrogen bonds were formed between compound **8g** and DDR2, including the conventional hydrogen bond between the pyrimidine nitrogen and Gln711 (2.37 Å), and aromatic hydrogen bonds involving Asp708 and the p-fluorophenyl group (2.27 Å), as well as Leu616 and the p-bromophenyl group (2.71 Å) (Figure 5A,B). Over 20 amino acid residues, including Arg780 and Tyr783, contributed to hydrophobic interactions. The elongated structure of compound **8g** facilitated optimal alignment within the DDR2 binding cavity, thereby inhibiting its activity. These results suggest that compound **8g** provided a thorough understanding of its interactions with the DDR2 active site. This could pave the way for further development and optimization of DDR-targeting drugs, contributing to advancements in cancer therapeutics.

#### In Slico ADME Studies of Compounds **8f** and **8g**

We evaluated the in silico absorption, distribution, metabolism, and excretion (ADME) properties of compounds **8f** and **8g** using the QikProp module from Schrödinger [66] (Table 3). The assessment revealed that both compounds have acceptable molecular weights and conform to Lipinski’s rule of five [67]. However, each compound violates one aspect of this rule due to its elevated lipophilicity, with QPlogPo/w values of 6.95 and 7.19, respectively. Both compounds lack hydrogen bond donors and have a moderate number of hydrogen bond acceptors (2.5 each) yet demonstrate excellent passive permeability. Their small polar surface area (PSA) of 24.41 Å^2^ promotes efficient membrane diffusion, as evidenced by their high Caco-2 permeability values (7667.0 for **8f** and 10,000.0 for **8g**). These findings suggest strong potential for gastrointestinal absorption and the ability to penetrate the blood–brain barrier (BBB) [68]. The estimated human oral absorption for both compounds is excellent (100%), as supported by binary prediction values of 1.

Although high lipophilicity enhances absorption, it hampers water solubility. QPlogS values of −8.35 and −8.73 for **8f** and **8g**, respectively, are significantly below the ideal threshold of −5.0, indicating poor aqueous solubility. Regarding skin permeability, QPlogKp values indicate moderate permeability (−0.180 for **8f** and −0.202 for **8g**), which reduces the relevance for systemic delivery. Regarding distribution, the predicted plasma protein binding (QPlogKhsa) was also moderate, with values of 1.540 and 1.587, respectively, suggesting balanced tissue exposure. Both compounds are anticipated to undergo a single metabolic reaction (#metab = 1), which is indicative of metabolic stability and a lower risk of rapid clearance. However, the predicted QPlogHERG values of −6.218 and −6.094, respectively, raise a notable safety concern as values below −5 may signal potential cardiotoxicity associated with hERG K^+^ channel inhibition. Further investigation is necessary during optimization efforts. In summary, despite certain limitations, particularly in solubility and potential cardiotoxicity, both compounds present favorable ADME profiles.

## 3. Experimental Section

### 3.1. Chemistry

#### 3.1.1. Materials and Methods

Chemical reagents were purchased from Adamas-beta (Shanghai, China) and Sigma-Aldrich (Merck Chemicals Co., Ltd., Shanghai, China). Visualization was performed using 254 nm UV light, and column chromatography was conducted with 200–300 mesh silica gel (Qingdao Haiyang Chemical Co., Qingdao, China), eluent petroleum ether/ethyl acetate (3:1). Melting points were determined using a Buchi B-540 melting point apparatus (BÜCHI Labortechnik AG, Flawil, Switzerland). The high-resolution mass spectra (HRMS) were recorded with AB SCIEX QSTAR Elite quadrupole time-of-flight mass spectrometry (AB SCIEX LLC, MA, USA). For ^1^H and ^13^C nuclear magnetic resonance (NMR) spectra, TMS was used as an internal standard and they were recorded using a Varian 400 and 600 MHz NMR spectrometer (Varian, Inc., Palo Alto, CA, USA) in CDCl_3_ and DMSO-D_6_.

#### 3.1.2. General Synthetic Procedures for **6a** and **6b**

A solution of 2-amino pyrroles (**4a** and **4b**, 10 mmol) and lactams (12 mmol) in dry dioxane (20 mL) was cooled to between 0 and 5 °C. Then, phosphorus oxychloride (POCl_3_; 2.3 mL, 25 mmol) was added dropwise, and the mixture was refluxed for one hour. After evaporating the solvent and excess POCl_3_ under reduced pressure, the resulting solid was suspended in dichloromethane (DCM; 100 mL). Then, 10% aqueous ammonia was added to adjust the pH to 9. The mixture was extracted twice with DCM (30 mL each time). The organic phase was washed with brine, dried over anhydrous sodium sulfate, filtered, and concentrated to yield a crude product. Purification by silica gel chromatography produced pure compounds **6a** and **6b** [29].

2-(4-Chlorophenyl)-1-methyl-6,7,8,9-tetrahydropyrido[1,2-*a*]pyrrolo[2,3-*d*]pyrimidin-4(1*H*)-one (**6a**). Yield 31%, yellow solid, m.p.171–173 °C. ^1^H NMR (600 MHz, CDCl_3_) *δ* 7.42–7.36 (m, 4H), 6.67 (s, 1H), 4.09 (t, *J* = 6.2 Hz, 2H), 3.69 (s, 3H), 2.97 (t, *J* = 6.7 Hz, 2H), 1.97 (p, *J* = 6.1 Hz, 2H), 1.92 (p, *J* = 6.3 Hz, 2H); ^13^C NMR (101 MHz, CDCl_3_) *δ* 159.13, 153.94, 148.10, 135.31, 133.92, 130.51, 129.95, 128.85, 105.90, 102.01, 41.59, 31.81, 30.07, 22.26, 19.37; HRMS (ESI): calcd for C_17_H_17_ClN_3_O [M + H]^+^: 314.1060, found 314.1058.

2-(4-Bromophenyl)-1-methyl-1,6,7,8,9,10-hexahydro-4*H*-pyrrolo[2’,3’:4,5]pyrimido[1,2-*a*]azepin-4-one (**6b**). Yield 71%, yellow solid, m.p.176–177 °C. ^1^H NMR (600 MHz, CDCl_3_) *δ* 7.56 (d, *J* = 8.4 Hz, 2H), 7.32 (d, *J* = 8.4 Hz, 2H), 6.67 (s, 1H), 4.40 (s, 2H), 3.70 (s, 3H), 3.05 (s, 2H), 1.83 (s, 4H), 1.76 (s, 2H); ^13^C NMR (101 MHz, CDCl_3_) *δ* 158.99, 158.75, 147.95, 135.48, 131.82, 130.96, 130.22, 122.09, 105.69, 102.31, 42.21, 37.62, 30.02, 29.63, 27.99, 25.53; HRMS (ESI): calcd for C_18_H_19_BrN_3_O [M + H]^+^: 372.0711, found 372.0710.

#### 3.1.3. Synthetic Procedure for the Pyrrolo[2,3-*d*]pyrimidines **8a**–**j**

Derivatives (**6a** or **6b**) (5.0 mmol) and 2-OMe-Py (5.5 mmol) were added to anhydrous DCM. The reaction mixture was stirred for ten minutes at 0 °C. Next, 10 mmol of Tf_2_O was slowly added dropwise and stirred for 1 h. Next, the appropriate aromatic amines (**7a**–**e**) (10 mmol) were added. The reaction mixture was monitored by thin layer chromatography (TLC). The reaction mixture was washed with an aqueous solution of NaHCO_3_ and brine. The organic layer was then dried over anhydrous Na_2_SO_4_ and filtered. The filtrate was evaporated under reduced pressure, and the residue was purified by chromatography on a silica gel column to give the compounds **8a**–**j**.

(*E*)-2-(4-Chlorophenyl)-1-methyl-*N*-phenyl-6,7,8,9-tetrahydropyrido[1,2-*a*]pyrrolo[2,3-*d*]pyrimidin-4(1*H*)-imine (**8a**). Yield 99%, yellow solid, m.p. 281–283. ^1^H NMR (400 MHz, CDCl_3_) *δ* 7.32–7.27 (m, 3H), 7.26 (d, *J* = 1.7 Hz, 1H), 7.16–7.11 (m, 2H), 7.06–7.00 (m, 1H), 6.95–6.90 (m, 2H), 5.10 (s, 1H), 4.16 (t, *J* = 6.2 Hz, 2H), 3.61 (s, 3H), 2.95 (t, *J* = 6.7 Hz, 2H), 2.03 (p, *J* = 6.1 Hz, 2H), 1.93 (p, *J* = 6.4 Hz, 2H). ^13^C NMR (101 MHz, CDCl_3_) *δ* 153.96, 151.70, 148.58, 145.48, 133.31, 132.66, 130.64, 129.63, 128.99, 128.65, 122.16, 122.10, 104.14, 101.84, 43.88, 32.51, 29.83, 22.85, 19.41. HRMS (ESI): calcd for C_23_H_22_ClN_4_ [M + H]^+^: 389.1533, found: 389.1502.

(*E*)-2-(4-Chlorophenyl)-*N*-(4-fluorophenyl)-1-methyl-6,7,8,9-tetrahydropyrido[1,2-*a*]pyrrolo[2,3-*d*]pyrimidin-4(1*H*)-imine (**8b**). Yield 48%, yellow solid, m.p. 240–242. ^1^H NMR (400 MHz, CDCl_3_) *δ* 7.35–7.30 (m, 2H), 7.18–7.14 (m, 2H), 7.02–6.95 (m, 2H), 6.90–6.83 (m, 2H), 5.16 (s, 1H), 4.14 (t, *J* = 6.2 Hz, 2H), 3.62 (s, 3H), 2.95 (t, *J* = 6.7 Hz, 2H), 2.03 (p, *J* = 6.0 Hz, 2H), 1.93 (p, *J* = 6.4 Hz, 2H). ^13^C NMR (101 MHz, CDCl_3_) *δ* (160.03, 157.65, d, *J* = 239.5 Hz), 153.94, 149.13, 145.53, 133.51, 132.91, 130.48, 129.69, 128.73, 123.14, 115.68, 115.46, 103.85, 101.74, 43.98, 32.50, 29.83, 22.83, 19.36. HRMS (ESI): calcd for C_23_H_21_ClFN_4_ [M + H]^+^: 407.1439, found: 407.1408.

(*E*)-*N*,2-*Bis*(4-chlorophenyl)-1-methyl-6,7,8,9-tetrahydropyrido[1,2-*a*]pyrrolo[2,3-*d*]pyrimidin-4(1*H*)-imine (**8c**). Yield 68%, yellow solid, m.p. 273–275. ^1^H NMR (400 MHz, CDCl_3_) *δ* 7.36–7.31 (m, 2H), 7.27–7.21 (m, 2H), 7.20–7.14 (m, 2H), 6.89–6.83 (m, 2H), 5.26 (s, 1H), 4.13 (t, *J* = 6.2 Hz, 2H), 3.61 (s, 3H), 2.95 (t, *J* = 6.7 Hz, 2H), 2.03 (p, *J* = 6.2 Hz, 2H), 1.93 (p, *J* = 6.1 Hz, 2H). ^13^C NMR (101 MHz, CDCl_3_) *δ* 153.94, 150.35, 148.65, 145.53, 133.57, 133.00, 130.46, 129.77, 128.97, 128.75, 126.96, 123.41, 103.86, 101.67, 43.93, 32.49, 29.85, 22.82, 19.37. HRMS (ESI): calcd for C_23_H_21_Cl_2_N_4_ [M + H]^+^: 423.1143, found: 423.1115.

(*E*)-*N*-(4-Bromophenyl)-2-(4-chlorophenyl)-1-methyl-6,7,8,9-tetrahydropyrido[1,2-*a*]pyrrolo[2,3-*d*]pyrimidin-4(1*H*)-imine (**8d**). Yield 99%, yellow solid, m.p. 262–264. ^1^H NMR (400 MHz, CDCl_3_) *δ* 7.41–7.31 (m, 4H), 7.20–7.15 (m, 2H), 6.84–6.79 (m, 2H), 5.27 (s, 1H), 4.13 (t, *J* = 6.2 Hz, 2H), 3.61 (s, 3H), 2.95 (t, *J* = 6.7 Hz, 2H), 2.02 (p, *J* = 6.2 Hz, 2H), 1.93 (p, *J* = 6.1 Hz, 2H). ^13^C NMR (101 MHz, CDCl_3_) *δ* 153.94, 150.86, 148.52, 145.53, 133.58, 133.03, 131.89, 130.45, 129.79, 128.75, 123.91, 114.50, 103.85, 101.65, 43.92, 32.48, 29.82, 22.82, 19.37. HRMS (ESI): calcd for C_23_H_21_BrClN_4_ [M + H]^+^: 467.0638, found: 467.0607.

(*E*)-2-(4-Chlorophenyl)-1-methyl-*N*-(4-(trifluoromethyl)phenyl)-6,7,8,9-tetrahydropyrido[1,2-*a*]pyrrolo[2,3-*d*]pyrimidin-4(1*H*)-imine (**8e**). Yield 61%, yellow solid, m.p. 202–204. ^1^H NMR (400 MHz, CDCl_3_) *δ* 7.54 (d, *J* = 8.7 Hz, 2H), 7.36–7.31 (m, 2H), 7.18–7.13 (m, 2H), 7.04 (d, *J* = 8.5 Hz, 2H), 5.20 (s, 1H), 4.19 (t, *J* = 6.2 Hz, 2H), 3.63 (s, 3H), 2.99 (t, *J* = 6.7 Hz, 2H), 2.05 (p, *J* = 6.2 Hz, 2H), 1.95 (p, *J* = 6.3 Hz, 2H). ^13^C NMR (101 MHz, CDCl_3_) *δ* 153.90, 148.51, 145.94, 133.85, 133.83, 130.13, 129.69, 128.81, (126.24, 126.21, 126.17, 126.13, q, *J* = 3.8 Hz), 124.47, 124.15, 123.36, 122.51, 122.45, 103.50, 101.64, 44.41, 32.47, 29.89, 22.76, 19.27. HRMS (ESI): calcd for C_24_H_21_ClF_3_N_4_ [M + H]^+^: 457.1407, found: 457.1374.

(*E*)-2-(4-Bromophenyl)-1-methyl-*N*-phenyl-1,6,7,8,9,10-hexahydro-4*H*-pyrrolo[2’,3’:4,5]pyrimido[1,2-*a*]azepin-4-imine (**8f**). Yield 45%, yellow solid, m.p. 188–190. ^1^H NMR (400 MHz, CDCl_3_) *δ* 7.52–7.47 (m, 2H), 7.38–7.32 (m, 2H), 7.24–7.15 (m, 3H), 7.09–7.04 (m, 2H), 5.06 (s, 1H), 4.65–4.59 (m, 2H), 3.65 (s, 3H), 3.21–3.15 (m, 2H), 2.02–1.94 (m, 2H), 1.88 (s, 4H). ^13^C NMR (101 MHz, CDCl_3_) *δ* 158.76, 149.66, 147.17, 131.92, 131.84, 130.24, 130.13, 129.80, 129.35, 125.63, 124.65, 122.72, 118.80, 103.64, 101.73, 47.18, 37.63, 30.06, 29.04, 27.23, 25.31. HRMS (ESI): calcd for C_24_H_24_BrN_4_ [M + H]^+^: 447.1184, found: 447.1150.

(*E*)-2-(4-Bromophenyl)-*N*-(4-fluorophenyl)-1-methyl-1,6,7,8,9,10-hexahydro-4*H*-pyrrolo[2’,3’:4,5]pyrimido[1,2-*a*]azepin-4-imine (**8g**). Yield 72%, yellow solid, m.p. 210-212. ^1^H NMR (400 MHz, CDCl_3_) *δ* 7.59–7.54 (m, 2H), 7.37–7.30 (m, 2H), 7.15–7.07 (m, 4H), 5.12 (s, 1H), 4.67–4.62 (m, 2H), 3.70 (s, 3H), 3.31–3.25 (m, 2H), 2.10–2.03 (m, 2H), 1.92 (s, 4H). ^13^C NMR (101 MHz, CDCl_3_) *δ* (162.86, 160.40, d, *J* = 248.0 Hz), 158.61, 150.52, 148.03, 132.15, 130.31, 129.01, 127.85, 123.55, 121.94, 116.56, 116.33, 102.96, 101.50, 48.77, 37.52, 30.19, 28.73, 27.05, 25.10. HRMS (ESI): calcd for C_24_H_23_BrFN_4_ [M + H]^+^: 465.1090, found: 465.1056.

(*E*)-2-(4-Bromophenyl)-*N*-(4-chlorophenyl)-1-methyl-1,6,7,8,9,10-hexahydro-4*H*-pyrrolo[2’,3’:4,5]pyrimido[1,2-*a*]azepin-4-imine (**8h**). Yield 61%, yellow solid, m.p. 240–242. ^1^H NMR (400 MHz, CDCl_3_) *δ* 7.52–7.47 (m, 2H), 7.25–7.21 (m, 2H), 7.13–7.08 (m, 2H), 6.87–6.82 (m, 2H), 5.24 (s, 1H), 4.59–4.53 (m, 2H), 3.61 (s, 3H), 3.06–3.01 (m, 2H), 1.91–1.78 (m, 6H). ^13^C NMR (101 MHz, CDCl_3_) *δ* 159.03, 148.30, 145.57, 133.15, 131.71, 130.87, 130.05, 128.98, 126.91, 123.35, 121.74, 104.05, 101.58, 44.19, 37.81, 29.83, 29.57, 27.41, 25.63. HRMS (ESI): calcd for C_24_H_23_BrClN_4_ [M + H]^+^: 481.0795, found: 481.0763.

(*E*)-*N*,2-*Bis*(4-bromophenyl)-1-methyl-1,6,7,8,9,10-hexahydro-4*H*-pyrrolo[2’,3’:4,5]pyrimido[1,2-*a*]azepin-4-imine (**8i**). Yield 77%, yellow solid, m.p. 247–249. ^1^H NMR (400 MHz, CDCl_3_) *δ* 7.52–7.47 (m, 2H), 7.40–7.35 (m, 2H), 7.12–7.08 (m, 2H), 6.83–6.78 (m, 2H), 5.25 (s, 1H), 4.59–4.53 (m, 2H), 3.61 (s, 3H), 3.06–3.01 (m, 2H), 1.90–1.79 (m, 7H). ^13^C NMR (101 MHz, CDCl_3_) *δ* 159.02, 150.61, 148.20, 145.60, 133.22, 131.91, 131.72, 130.85, 130.06, 123.90, 121.77, 114.49, 104.03, 101.57, 44.23, 37.81, 29.83, 29.57, 27.41, 25.63. HRMS (ESI): calcd for C_24_H_23_Br_2_N_4_ [M + H]^+^: 525.0289, found: 525.0256.

(*E*)-2-(4-Bromophenyl)-1-methyl-*N*-(4-(trifluoromethyl)phenyl)-1,6,7,8,9,10-hexahydro-4*H*-pyrrolo[2’,3’:4,5]pyrimido[1,2-*a*]azepin-4-imine (**8j**). Yield 95%, yellow solid, m.p. 188–190. ^1^H NMR (400 MHz, CDCl_3_) *δ* 7.52 (d, *J* = 8.3 Hz, 2H), 7.50–7.45 (m, 2H), 7.10–7.05 (m, 2H), 6.99 (d, *J* = 8.2 Hz, 2H), 5.19 (s, 1H), 4.60–4.54 (m, 2H), 3.62 (s, 3H), 3.05 (d, *J* = 5.7 Hz, 2H), 1.93–1.79 (m, 6H). ^13^C NMR (101 MHz, CDCl_3_) *δ* 159.00, 155.06, 147.94, 145.69, 133.41, 131.73, 130.76, 129.92, (126.20, 126.17, 126.13, 126.09, q, *J* = 3.5 Hz), 123.87, 123.55, 123.48, 122.10, 121.80, 103.87, 101.50, 44.28, 37.80, 29.84, 29.57, 27.40, 25.62. HRMS (ESI): calcd for C_25_H_23_BrF_3_N_4_ [M + H]^+^: 515.1058, found: 515.1024.

#### 3.1.4. Synthetic Procedure for the Pyrrolo[2,3-d]pyrimidines **10a**–**f**

A mixture of **6a** or **6b** (5.0 mmol) and NXS (**9a**–**c**) (5.5 mmol) in DCM was stirred at room temperature for 1 h. With **9a** (*N*-chlorosuccinimide), the reaction mixture was refluxed for 1 h. The reaction was monitored by TLC. The reaction mixture was washed (saturated aqueous NaHCO_3_ solution), the organic layer dried over anhydrous Na_2_SO_4,_ and filtered. The filtrate was evaporated under reduced pressure, and the residue was purified by chromatography on a silica gel column to give the compounds **10a**–**f**.

3-Chloro-2-(4-chlorophenyl)-1-methyl-6,7,8,9-tetrahydropyrido[1,2-*a*]pyrrolo[2,3-*d*]pyrimidin-4(1*H*)-one (**10a**). Yield 77%, yellow solid, m.p. 161–163. ^1^H NMR (400 MHz, C_6_D_6_) *δ* 7.15–7.10 (m, 2H), 7.04–6.98 (m, 2H), 3.71 (t, *J* = 6.1 Hz, 2H), 3.10 (s, 3H), 2.49 (t, *J* = 6.6 Hz, 2H), 1.22–1.09 (m, 4H). ^13^C NMR (101 MHz, C_6_D_6_) *δ* 157.09, 154.53, 145.92, 134.20, 131.62, 129.68, 128.57, 127.55, 105.83, 103.29, 41.00, 31.51, 29.24, 21.64, 18.82. HRMS (ESI): calcd for C_17_H_16_Cl_2_N_3_O [M + H]^+^: 348.0670, found: 348.0644.

3-Bromo-2-(4-chlorophenyl)-1-methyl-6,7,8,9-tetrahydropyrido[1,2-*a*]pyrrolo[2,3-*d*]pyrimidin-4(1*H*)-one (**10b**). Yield 56%, yellow solid, m.p. 135–136. ^1^H NMR (400 MHz, C_6_D_6_) *δ* 7.14–7.10 (m, 2H), 7.01–6.96 (m, 2H), 3.70 (t, *J* = 6.1 Hz, 2H), 3.10 (s, 3H), 2.48 (t, *J* = 6.6 Hz, 2H), 1.20–1.07 (m, 4H). ^13^C NMR (101 MHz, C_6_D_6_) *δ* 157.28, 154.38, 146.57, 134.31, 131.84, 131.56, 128.52, 128.28, 104.58, 90.88, 41.03, 31.52, 29.47, 21.63, 18.80. HRMS (ESI): calcd for C_17_H_16_BrClN_3_O [M + H]^+^: 392.0165, found: 392.0138.

2-(4-Chlorophenyl)-3-iodo-1-methyl-6,7,8,9-tetrahydropyrido[1,2-*a*]pyrrolo[2,3-*d*]pyrimidin-4(1*H*)-one (**10c**). Yield 75%, yellow solid, m.p. 168–169. ^1^H NMR (400 MHz, C_6_D_6_) *δ* 7.13–7.09 (m, 2H), 6.96–6.91 (m, 2H), 3.68 (t, *J* = 6.1 Hz, 2H), 3.11 (s, 3H), 2.48 (t, *J* = 6.6 Hz, 2H), 1.19–1.06 (m, 4H). ^13^C NMR (101 MHz, C_6_D_6_) *δ* 157.61, 154.06, 147.47, 135.44, 134.44, 132.15, 129.65, 128.52, 106.63, 56.76, 41.08, 31.58, 29.87, 21.63, 18.79. HRMS (ESI): calcd for C_17_H_16_ClIN_3_O [M + H]^+^: 440.0027, found: 439.9998.

2-(4-Bromophenyl)-3-chloro-1-methyl-1,6,7,8,9,10-hexahydro-4*H*-pyrrolo[2’,3’:4,5]pyrimido[1,2-*a*]azepin-4-one (**10d**). Yield 60%, yellow solid, m.p. 224–226. ^1^H NMR (400 MHz, CDCl_3_) *δ* 7.64–7.59 (m, 2H), 7.35–7.31 (m, 2H), 4.41–4.35 (m, 2H), 3.59 (s, 3H), 3.06–3.00 (m, 2H), 1.86–1.80 (m, 4H), 1.79–1.72 (m, 2H). ^13^C NMR (101 MHz,CDCl_3_) *δ* 159.70, 157.90, 145.84, 131.89, 131.83, 130.62, 127.74, 122.98, 105.81, 102.78, 41.99, 37.65, 30.13, 29.59, 27.88, 25.45. HRMS (ESI): calcd for C_18_H_18_BrClN_3_O [M + H]^+^: 406.0322, found: 406.0295.

3-Bromo-2-(4-bromophenyl)-1-methyl-1,6,7,8,9,10-hexahydro-4*H*-pyrrolo[2’,3’:4,5]pyrimido[1,2-*a*]azepin-4-one (**10e**). Yield 81%, yellow solid, m.p. 208–209. ^1^H NMR (400 MHz, CDCl_3_) *δ* 7.64–7.59 (m, 2H), 7.35–7.29 (m, 2H), 4.41–4.35 (m, 2H), 3.59 (s, 3H), 3.06–3.01 (m, 2H), 1.83 (s, 4H), 1.79–1.73 (m, 2H). ^13^C NMR (101 MHz, CDCl_3_) *δ* 159.61, 158.04, 146.42, 132.52, 132.10, 131.85, 131.82, 128.46, 123.12, 104.03, 90.89, 42.08, 37.65, 30.38, 29.60, 27.86, 25.45. HRMS (ESI): calcd for C_18_H_18_Br_2_N_3_O [M + H]^+^: 449.9817, found: 449.9785.

2-(4-Bromophenyl)-3-iodo-1-methyl-1,6,7,8,9,10-hexahydro-4*H*-pyrrolo[2’,3’:4,5]pyrimido[1,2-*a*]azepin-4-one (**10f**). Yield 97%, yellow solid, m.p. 209–211. ^1^H NMR (400 MHz, CDCl_3_) *δ* 7.65–7.60 (m, 2H), 7.31–7.27 (m, 2H), 4.41–4.35 (m, 2H), 3.59 (s, 3H), 3.06–3.00 (m, 2H), 1.87–1.80 (m, 4H), 1.79–1.73 (m, 2H). ^13^C NMR (101 MHz, CDCl_3_) *δ* 159.34, 158.33, 147.45, 136.41, 132.40, 131.86, 131.81, 129.84, 123.26, 106.05, 56.91, 42.16, 37.75, 30.72, 29.63, 27.88, 25.49. HRMS (ESI): calcd for C_18_H_18_BrIN_3_O [M + H]^+^: 497.9678, found: 497.9641.

### 3.2. Biology

#### Cytotoxic Activity Assay

The MCF-7, HeLa, and HT-29 cell lines, obtained from the Chinese Type Culture Collection, CAS (Shanghai, China), were used as models for cytotoxicity screening. The samples were dissolved in dimethyl sulfoxide (DMSO) (50 μM) and stored at 4 °C. Cell viability was determined using the MTT assay. For the MTT assay, the target cells were seeded in 96-well plates and treated with the synthesized samples for 72 h. Then, 5 mg/mL MTT was added to the culture medium, and 10 μL/well was added. The plate was then incubated in the dark at 37 °C for 4 h. The medium was removed, and 100 μL of dimethyl sulfoxide was added per well. OD570 was measured using Spectra Max M5 (Molecular Devices, CA, USA). Cell viability (%) = OD_compound_ /OD_DMSO_ × 100%. IC_50_ values were calculated using GraphPad Prism 9 (CA, USA) by nonlinear regression of the log-transformed data (X Log[X]), with the log (inhibitor) vs. normalized response-variable slope tool.

### 3.3. Molecular Docking

Docking simulations were conducted using Maestro version 12.8 from the 2021–2022 Schrödinger Suite [69,70]. The DDR2 crystal structure (PDB code 6FER, 2.87 Å resolution) was utilized from the RCSB Protein Data Bank (https://www.rcsb.org/structure/6FER, accessed on 9 May 2025). The Protein Preparation Wizard was used to add hydrogens and prepare the protein for ionization at pH 7.0. Missing residues were addressed using the Prime module, and water molecules beyond 5 Å were removed. Energy minimization was performed using the OPLS4 force field, followed by optimization with the LigPrep module. A grid box covering 12 Å from the centroid was defined for docking and was initially set based on co-crystallized ligands. We validated the docking protocol by redocking the bound ligand and subsequently docking compound **8g** into the DDR2 active site.

#### ADME Pharmacokinetic Analysis

The ADME properties of the compounds were assessed using the QikProp module within the Maestro interface (Schrödinger Release 2022-1: QikProp, Schrödinger, LLC, New York, NY, 2021) [66]. Properties such as absorption, distribution, metabolism, and excretion were calculated in an accurate prediction mode. We determined parameters related to drug-likeness and dynamics, including hydrogen bond donors and acceptors, Lipinski’s rule of five, the water–octanol partition coefficient, human serum albumin binding, the van der Waals surface area of polar nitrogen and oxygen atoms, calculated central nervous system activity, predicted IC_50_ value for HERG K^+^ ion channels, apparent Caco-2 cell permeability, Madin-Darby canine kidney cell line permeability, skin permeability, aqueous solubility, and percent of human oral absorption.

## 4. Conclusions

We have designed the modification of the pyrrolo[2,3-*d*]pyrimidine core with the tricyclic ring by synthesizing its imines via Tf_2_O/2-methoxypyridine-mediated carbonyl-amine condensation. This was followed by studies on the carbon-halogen bond formation at position C-3 of the pyrrole ring using *N*-halosuccinimides. All novel compounds were evaluated for their anticancer potential against three types of cancer cell lines: breast (MCF-7), cervical (HeLa), and colon (HT-29) cells, which are relevant cell models for anticancer drug discovery. Two pyrrolo[2,3-*d*]pyrimidine-imines containing a bromine substituent at position C-4 of the phenyl ring and pentamethylene (azepine) side-ring, **8f** and **8g**, exhibited significant cytotoxic activity on HT-29 cells with IC_50_ values of 4.55 ± 0.23 and 4.01 ± 0.20 µM, respectively. These results revealed an interesting pattern, where condensed pyrimidinones containing an azepine ring demonstrated selective antitumor activity on the colon cancer cell line HT-29. In addition, the molecular docking of compound **8g** with the DDR2 confirms its active site interactions. This could pave the way for further development and optimization of DDR-targeting drugs, contributing to advancements in cancer therapeutics. The physicochemical properties of the studied lead compounds need to be developed, especially their selectivity and water solubility, as these are essential for improving bioavailability in in vivo models. These compounds may serve as design templates for further studies.

## Figures and Tables

**Table 1:** In vitro cytotoxic activity of the synthesized pyrrolo[2,3-*d*]pyrimidines (**8a**–**j** and **10a**–**f**).

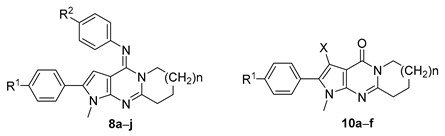							
Compd.	R_1_	R_2_	X	n ^a^	Cell Lines (IC_50_, µM)		
					HeLa	MCF-7	HT-29
**8a**	Cl	H	−	1	≥50	≥50	19.22 ± 0.91
**8b**	Cl	F	−	1	≥50	≥50	≥50
**8c**	Cl	Cl	−	1	≥50	≥50	≥50
**8d**	Cl	Br	−	1	≥50	≥50	≥50
**8e**	Cl	CF_3_	−	1	≥50	≥50	≥50
**8f**	Br	H	−	2	≥50	≥50	4.55 ± 0.23
**8g**	Br	F	−	2	≥50	≥50	4.01 ± 0.20
**8h**	Br	Cl	−	2	≥50	≥50	≥50
**8i**	Br	Br	−	2	≥50	≥50	≥50
**8j**	Br	CF_3_	−	2	≥50	≥50	≥50
**10a**	Cl	−	Cl	1	22.77 ± 0.98	27.52 ± 1.07	24.66 ± 1.13
**10b**	Cl	−	Br	1	≥50	20.05 ± 0.93	≥50
**10c**	Cl	−	I	1	≥50	≥50	34.16 ± 1.60
**10d**	Br	−	Cl	2	≥50	≥50	≥50
**10e**	Br	−	Br	2	≥50	≥50	15.97 ± 0.75
**10f**	Br	−	I	2	≥50	≥50	19.04 ± 0.92
**DOX**	−	−	−	−	0.94 ± 0.016	0.18 ± 0.011	0.82 ± 0.029

^a^ means the number of methylene (-CH_2_-) groups in the cycloalkane ring.

**Table 2:** IC_50_ values for human embryonic kidney (HEK-293) cells and SI values of **8f** and **8g** against the HT-29 cell line.

Compounds	IC_50_ (±SD, µM)	IC_50_ (HT-29), µM	SI^HT-29^
**8f**	195 ± 0.07	4.55 ± 0.23	42.86
**8g**	169 ± 0.10	4.01 ± 0.20	42.14

**Table 3:** In silico ADME properties of leads **8f** and **8g** by the QikProp module.

Parameters	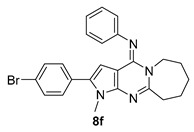	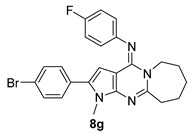
**Molecular weight ^a^**	447.376	465.367
**Rule of five ^b^**	1	1
**QPlogPo/w ^c^**	6.947	7.186
**QPlogS ^d^**	−8.349	−8.731
**QPlogHERG ^e^**	−6.218	−6.094
**QPPCaco ^f^**	7667.03	7667.03
**QPlogBB ^g^**	0.513	0.627
**Human oral absorption ^h^**	1	1

^a^ Molecular weight of the molecule (Range: 130.0–725.0). ^b^ Number of violations of Lipinski’s rule of five (Range: maximum is 4). ^c^ Predicted octanol/water partition coefficient (Range: −2.0–6.5). ^d^ Predicted aqueous solubility in mol dm-3 (Range: −6.5–0.5).^e^ Computed IC_50_ value for HERG K+ ion channels, concerning below −5. ^f^ Predicted apparent Caco-2 cell permeability (Range: <25 poor, >500 great). ^g^ Log BB, brain/blood partition coefficient (Range: −3.0–1.2). ^h^ Human oral absorption (Range: 1 = very low, 2 = moderate, 3 = high).

## Data Availability

The data that supports the structure confirmation of this study are available in the supplementary material of this article.

## Supplementary Materials

The following supporting information can be downloaded at: https://www.mdpi.com/article/10.3390/molecules30142917/s1, Figures S1–S48: The ^1^H and ^13^C NMR, along with the HRMS spectrum of compounds **8a**–**j** and **10a**–**f**.
