# Supplementary material for: Pyrrolopyrimidines: Design, Synthesis and Antitumor Properties of Novel Tricyclic Pyrrolo [2,3-d]pyrimidine Derivatives
Source: Molecules. 2025 Jul 10;30(14):2917. doi: 10.3390/molecules30142917 (PMC12299203; doi:10.3390/molecules30142917)
Supplement: Supplementary file 1 [file molecules-30-02917-s001.zip › molecules-3716245-supplementary.pdf]

SUPPLEMENTARY MATERIALS

Pyrrolopyrimidines: Design, Synthesis and Antitumor Properties of Novel Tricyclic Pyrrolo[2,3-*d*]pyrimidine Derivatives

^{1}H, ^{13}C NMR, and HRMS spectra of compounds **8a-j** and **10a-f**

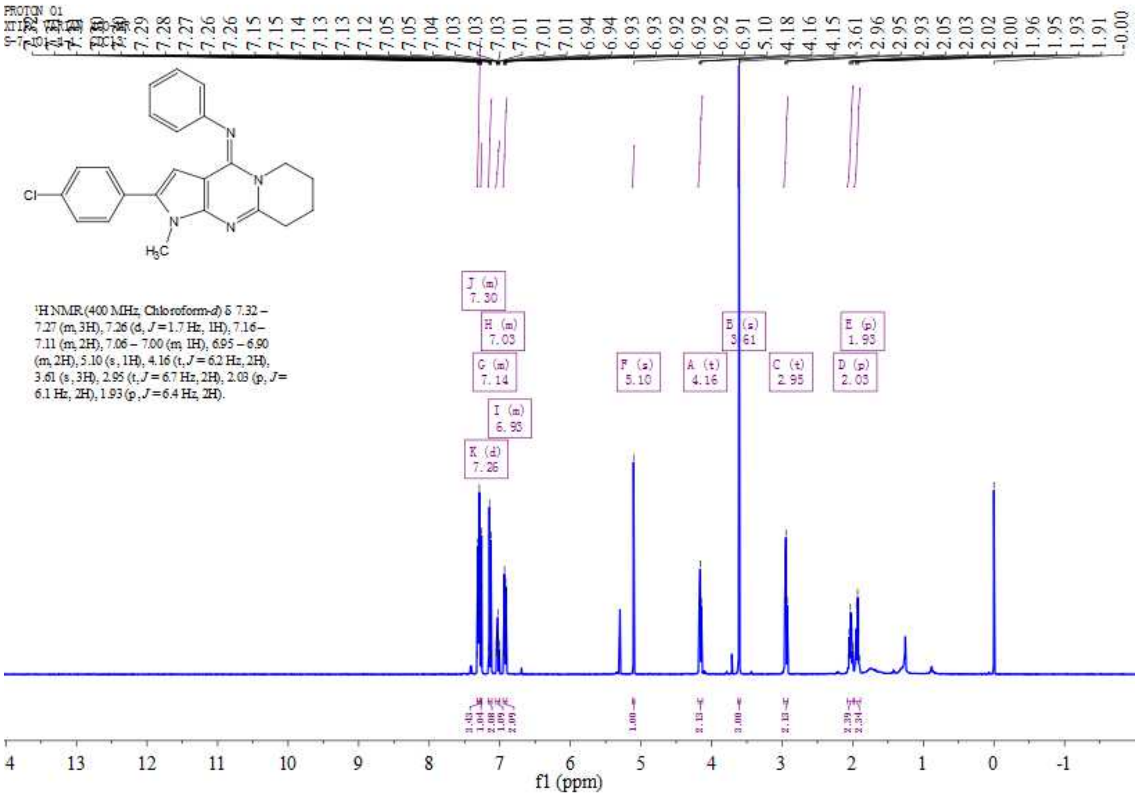

Figure S1. ^{1}H NMR spectrum of **8a**

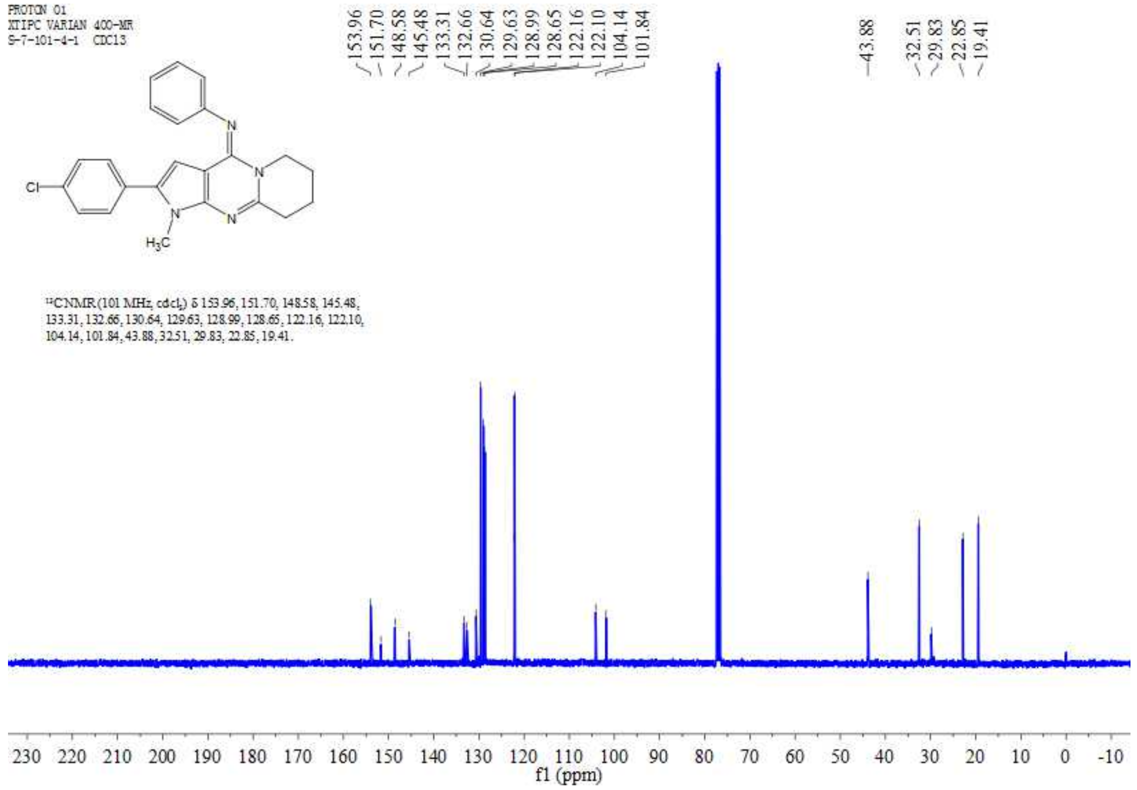

Figure S2. ^{13}C NMR spectrum of **8a**

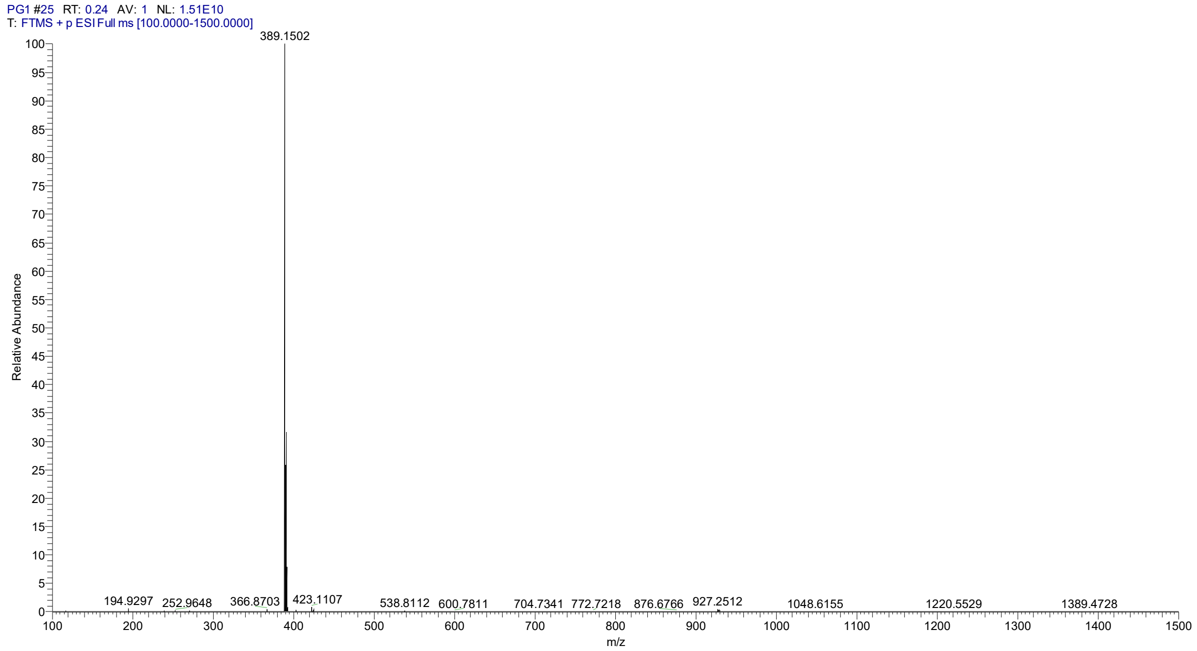

Figure S3. HRMS spectrum of **8a**

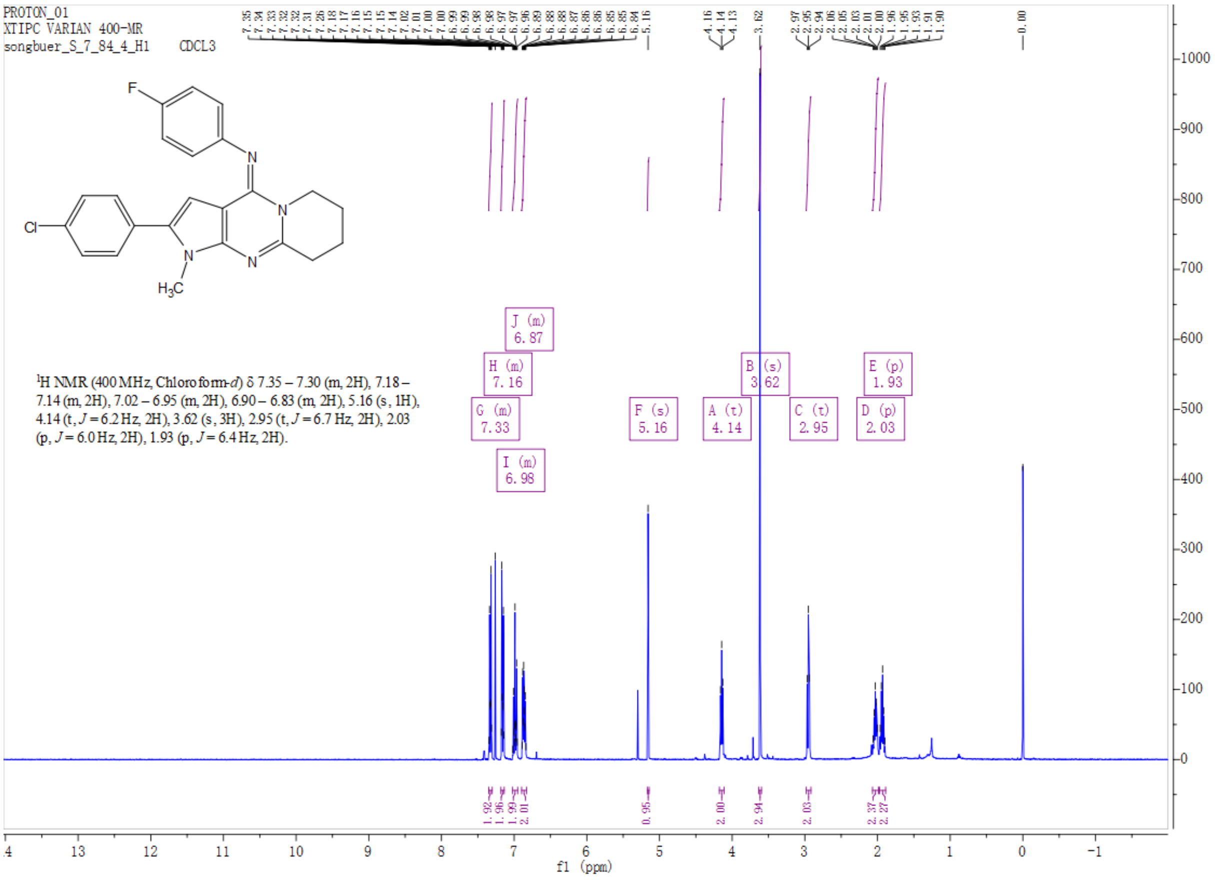

Figure S4. ^{1}H NMR spectrum of **8b**

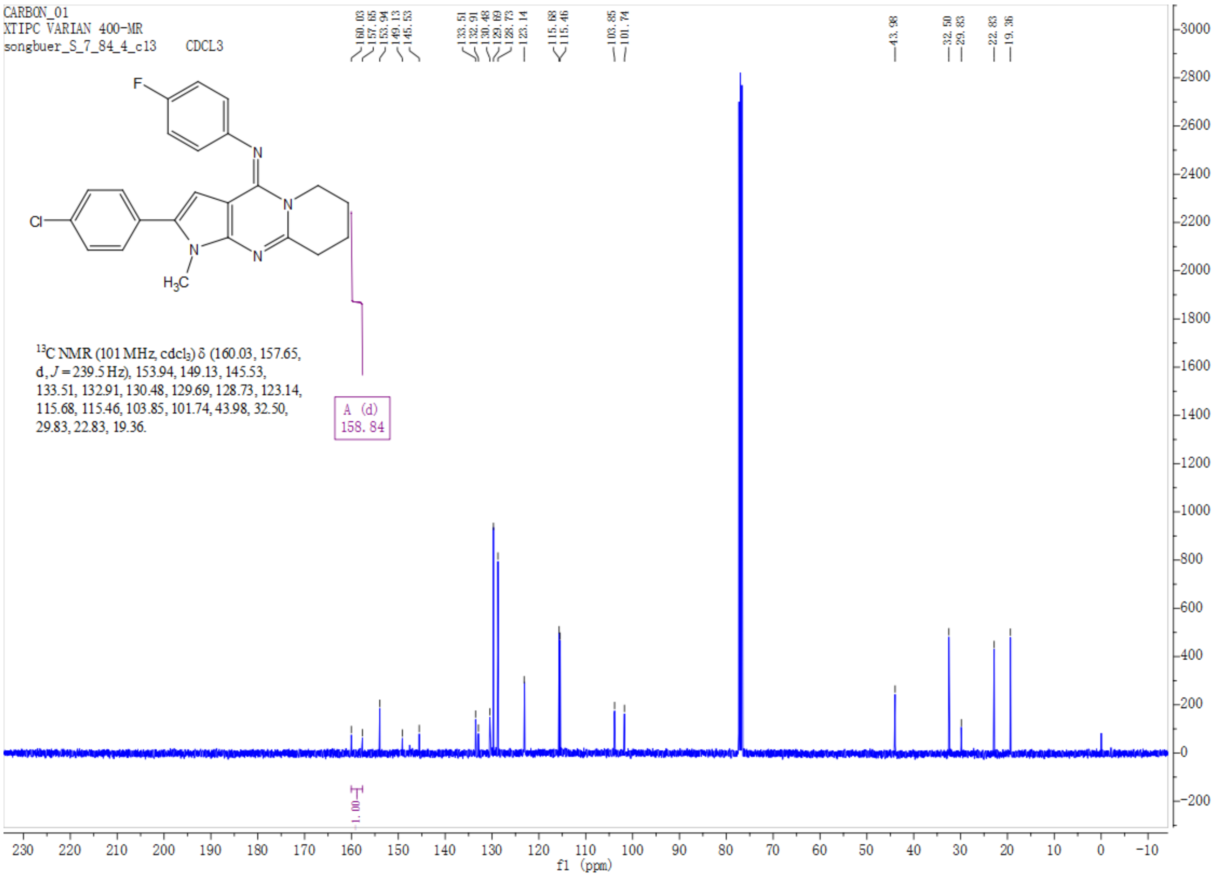

δ 160.03 and 157.65 are two peaks of the coupling splitting of a fluorine atom to carbon with a ratio of 1:1

Figure S5. ^{13}C NMR spectrum of **8b**

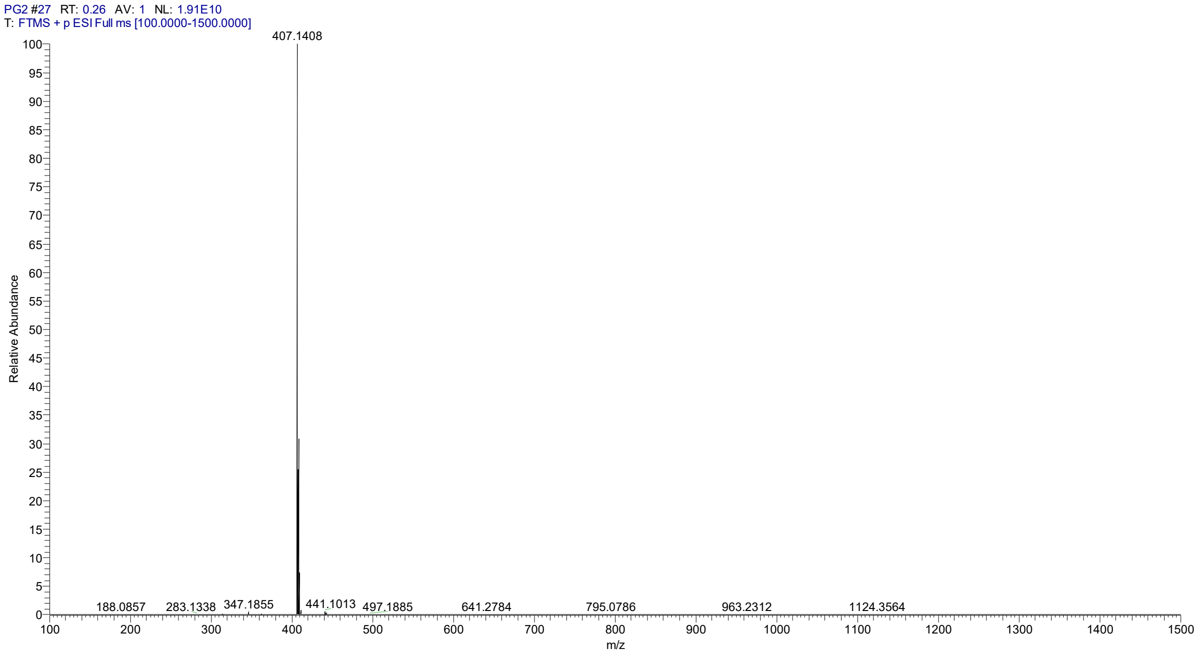

Figure S6. HRMS spectrum of **8b**

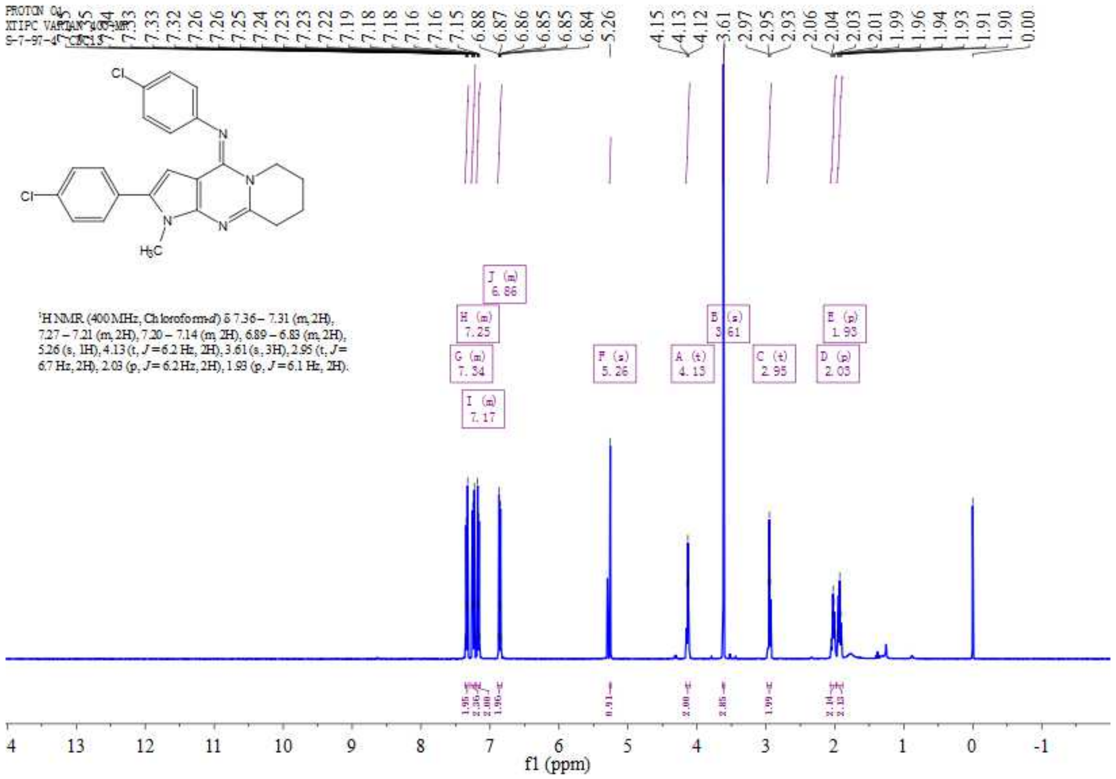

Figure S7. ^{1}H NMR spectrum of **8c**

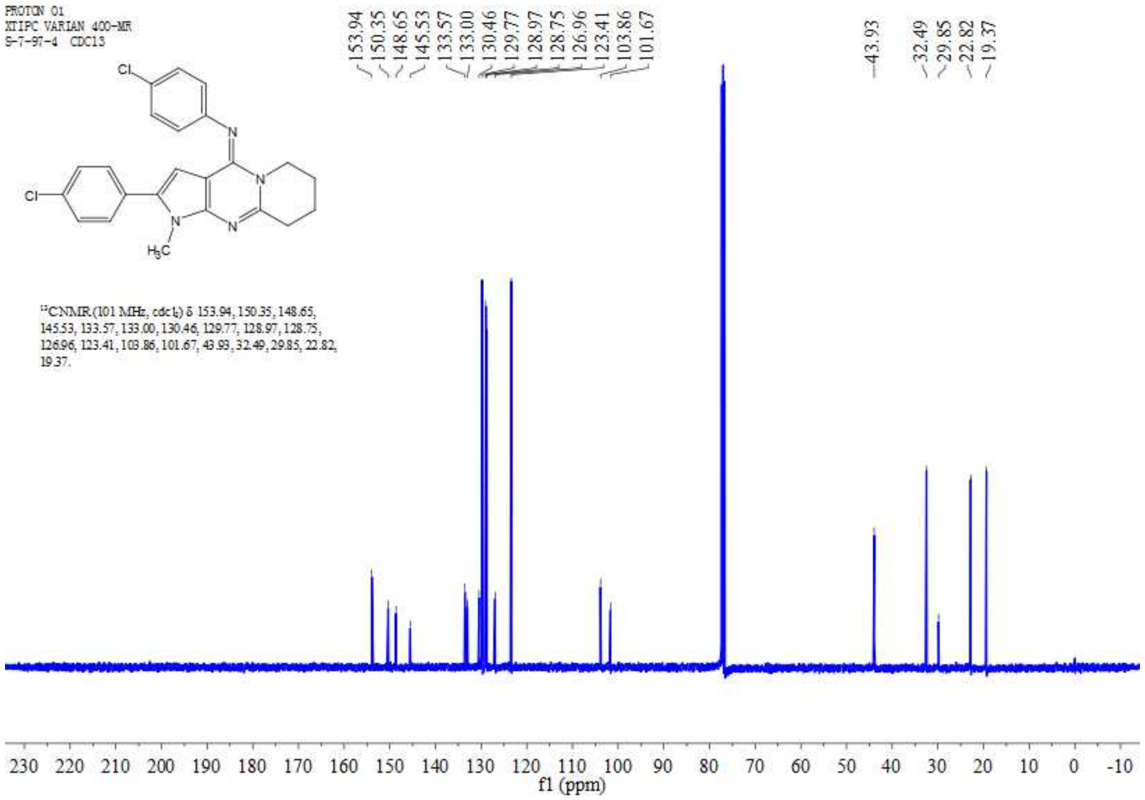

Figure S8. ^{13}C NMR spectrum of **8c**

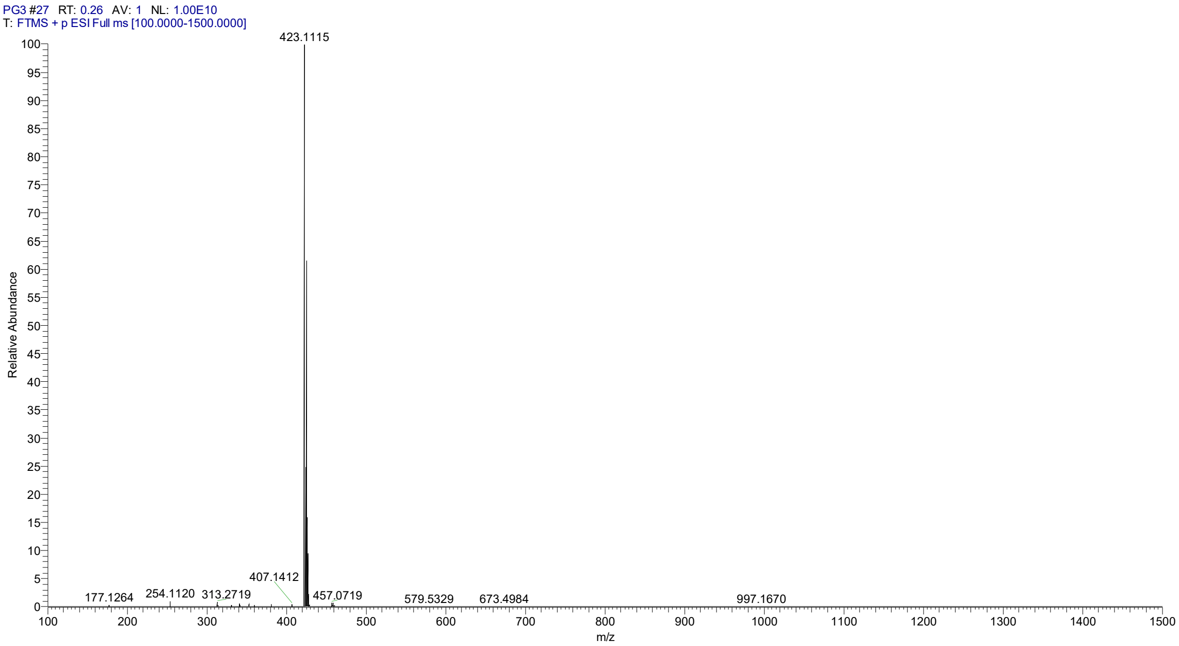

Figure S9. HRMS spectrum of **8c**

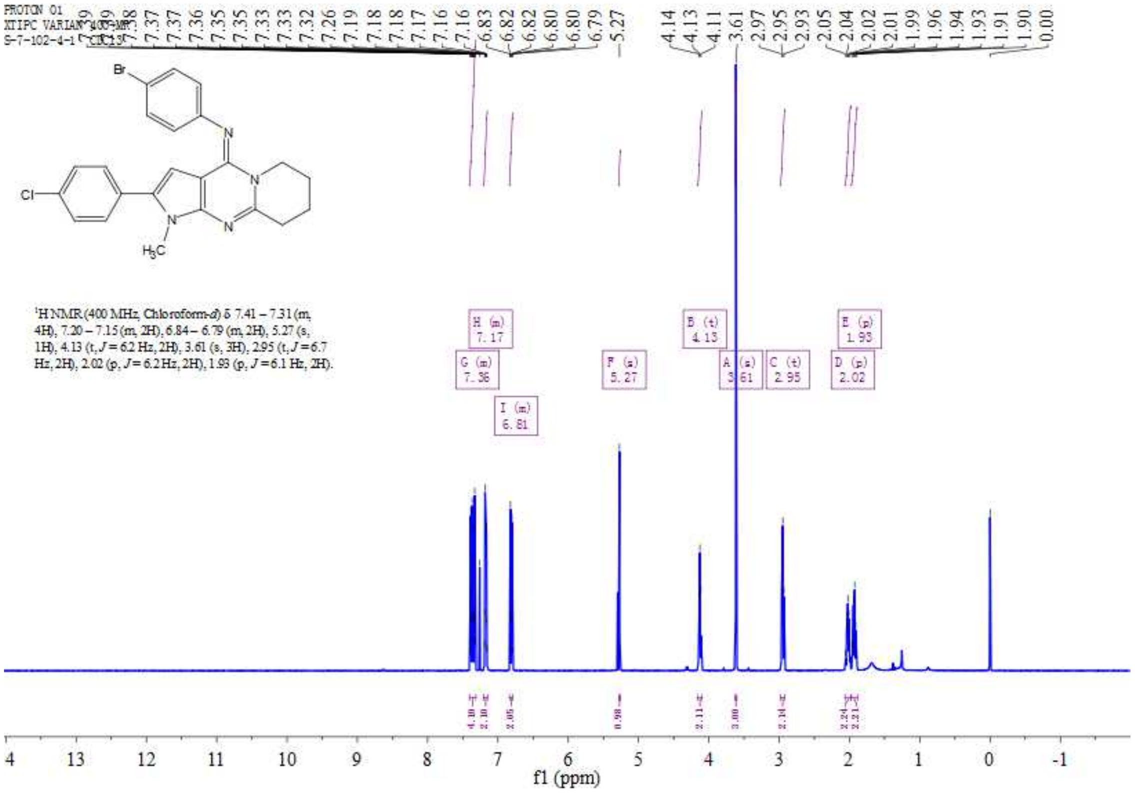

Figure S10. ^{1}H NMR spectrum of **8d**

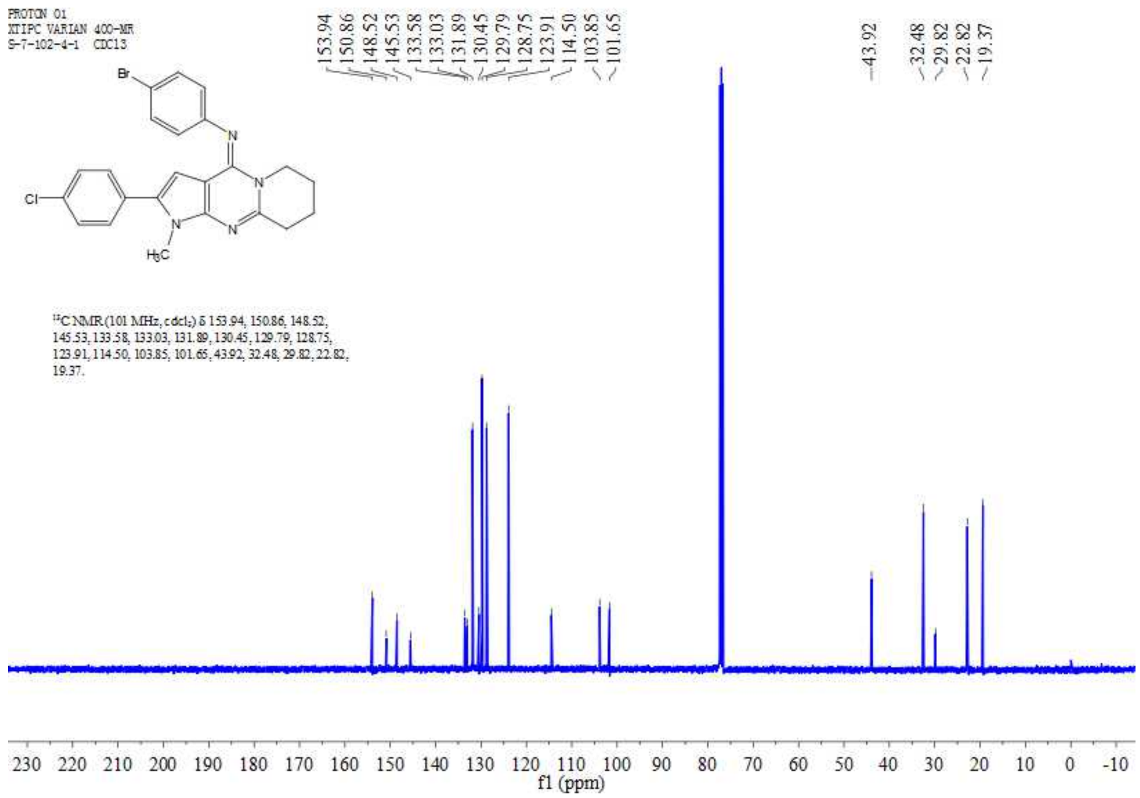

Figure S11. ^{13}C NMR spectrum of **8d**

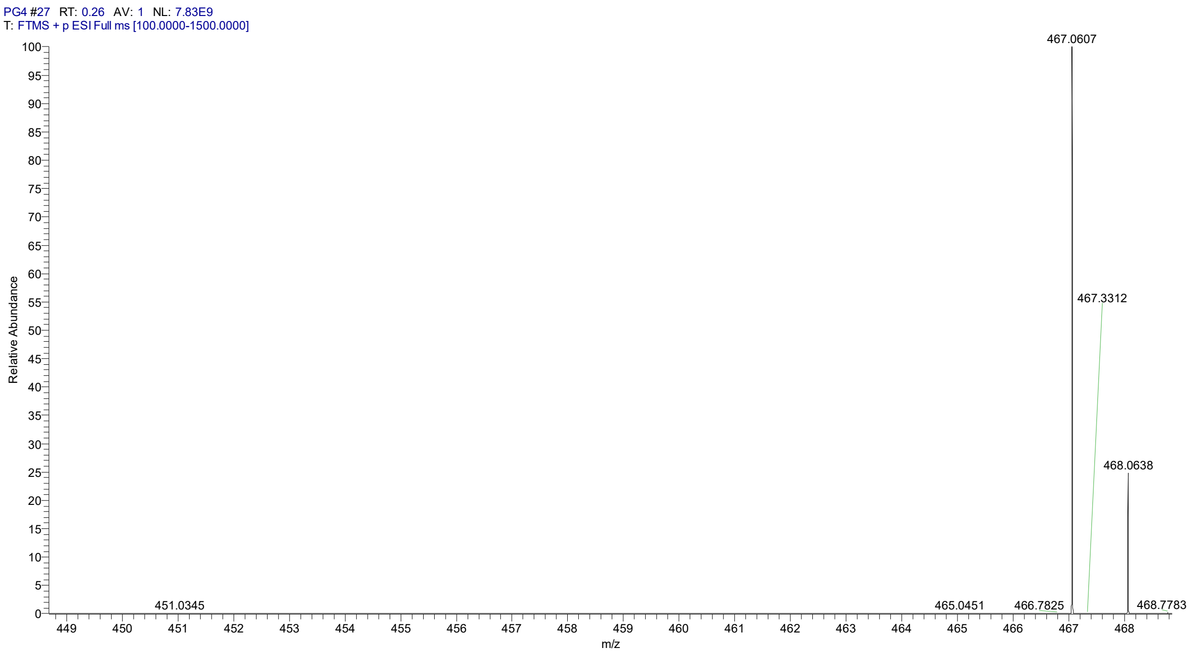

Figure S12. HRMS spectrum of **8d**

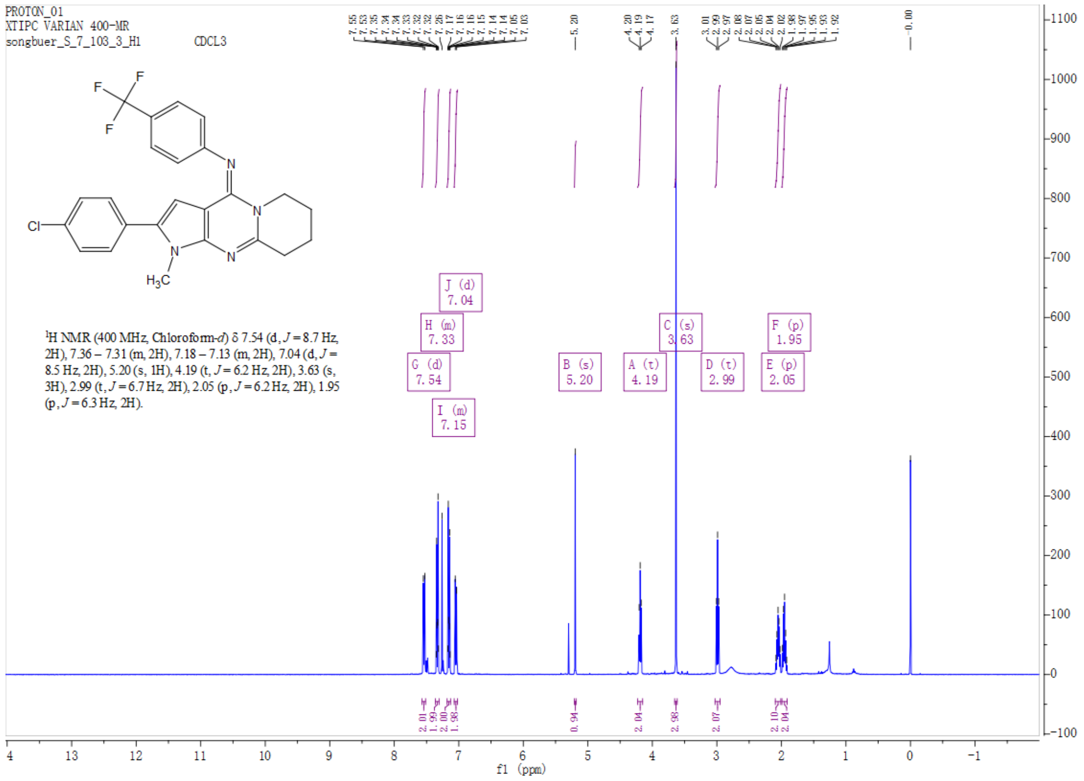

Figure S13. ^{1}H NMR spectrum of **8e**

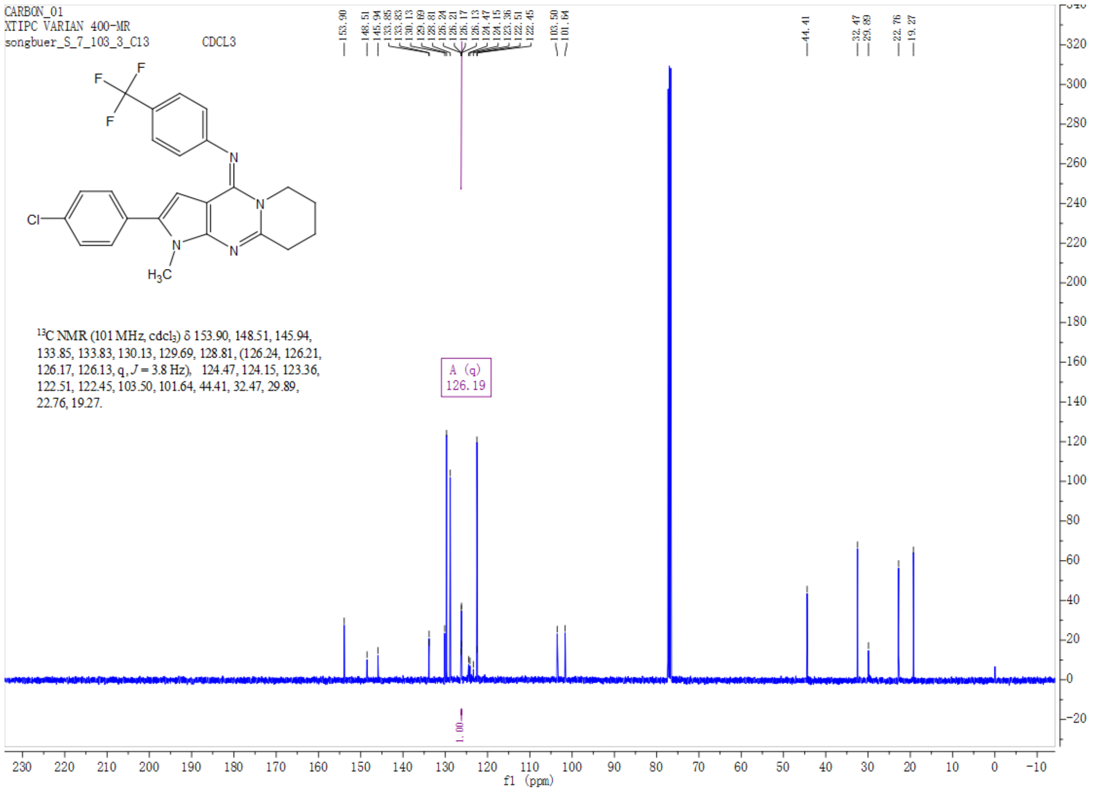

δ 126.24, 126.21, 126.17, 126.13 are the coupling splits of trifluoromethyl to carbon, the ratio is 1:3:3:1

Figure S14. ^{13}C NMR spectrum of **8e**

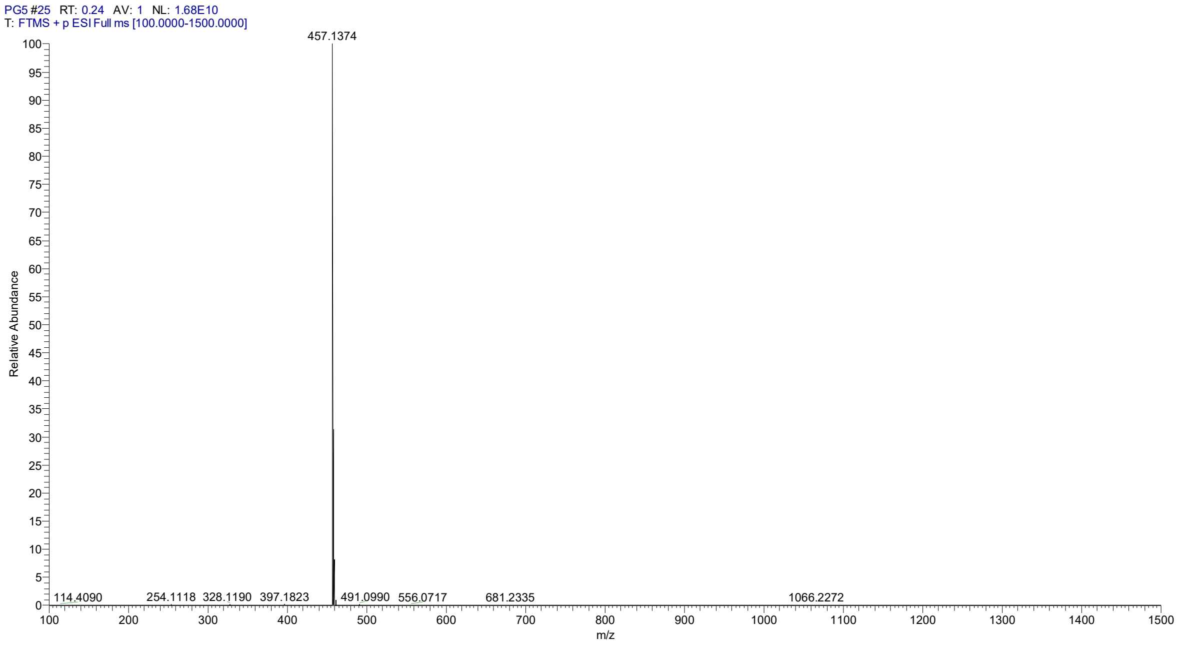

Figure S15. HRMS spectrum of **8e**

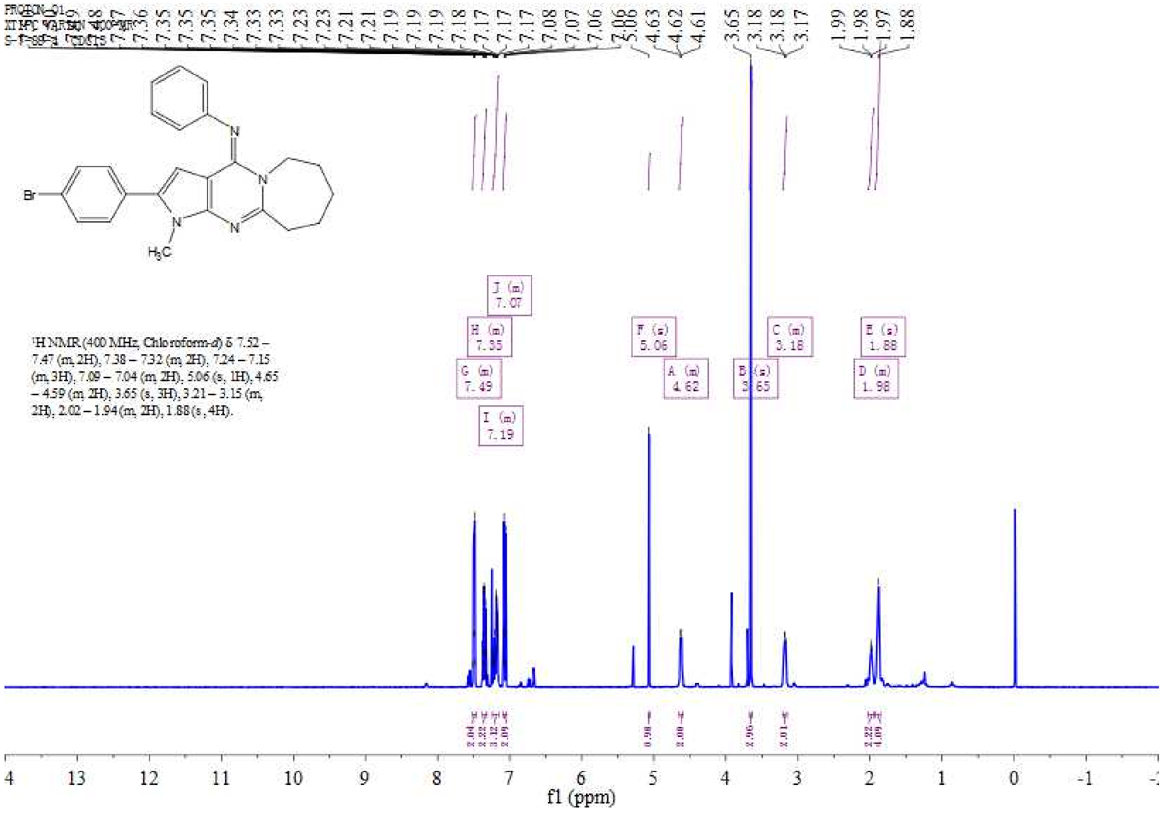

Figure S16. ^{1}H NMR spectrum of **8f**

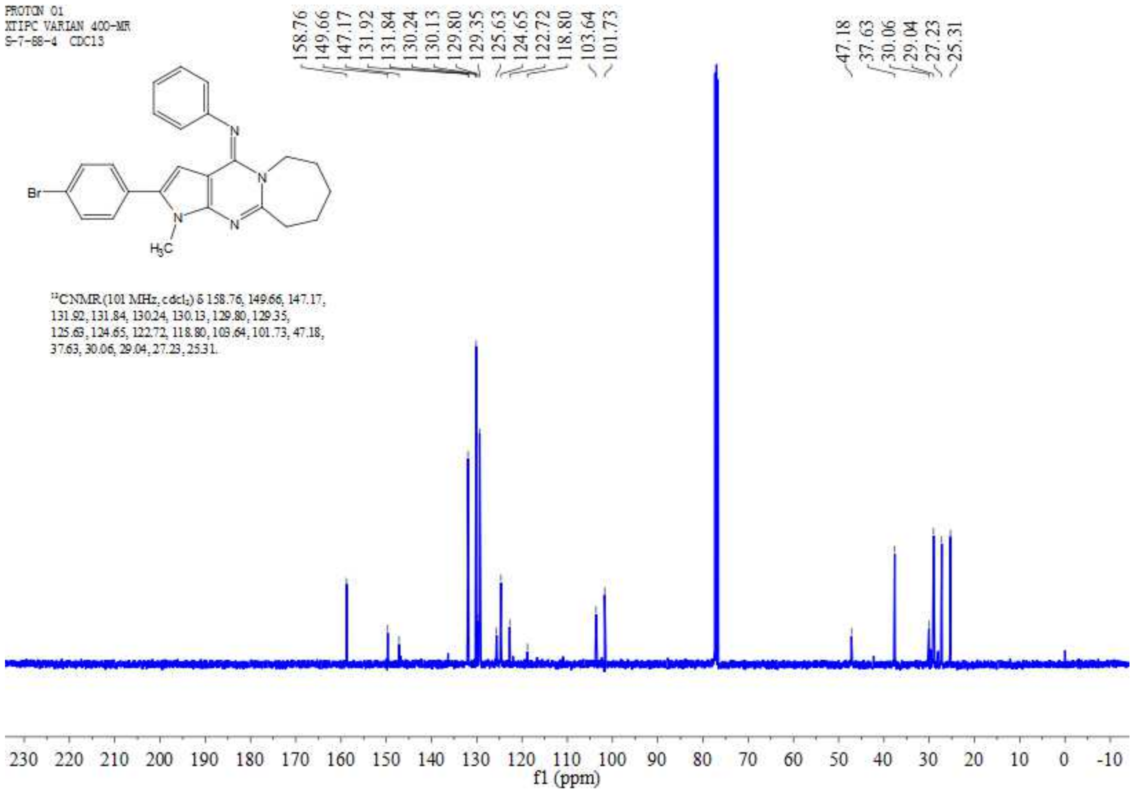

Figure S17. ^{13}C NMR spectrum of **8f**

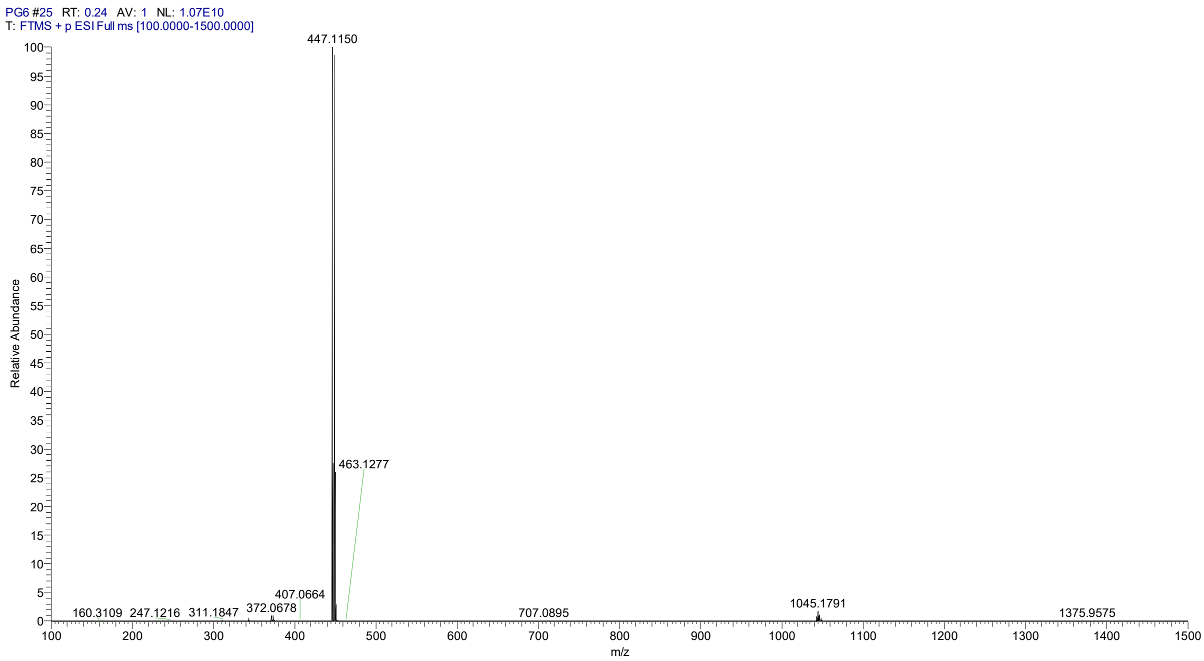

Figure S18. HRMS spectrum of **8f**

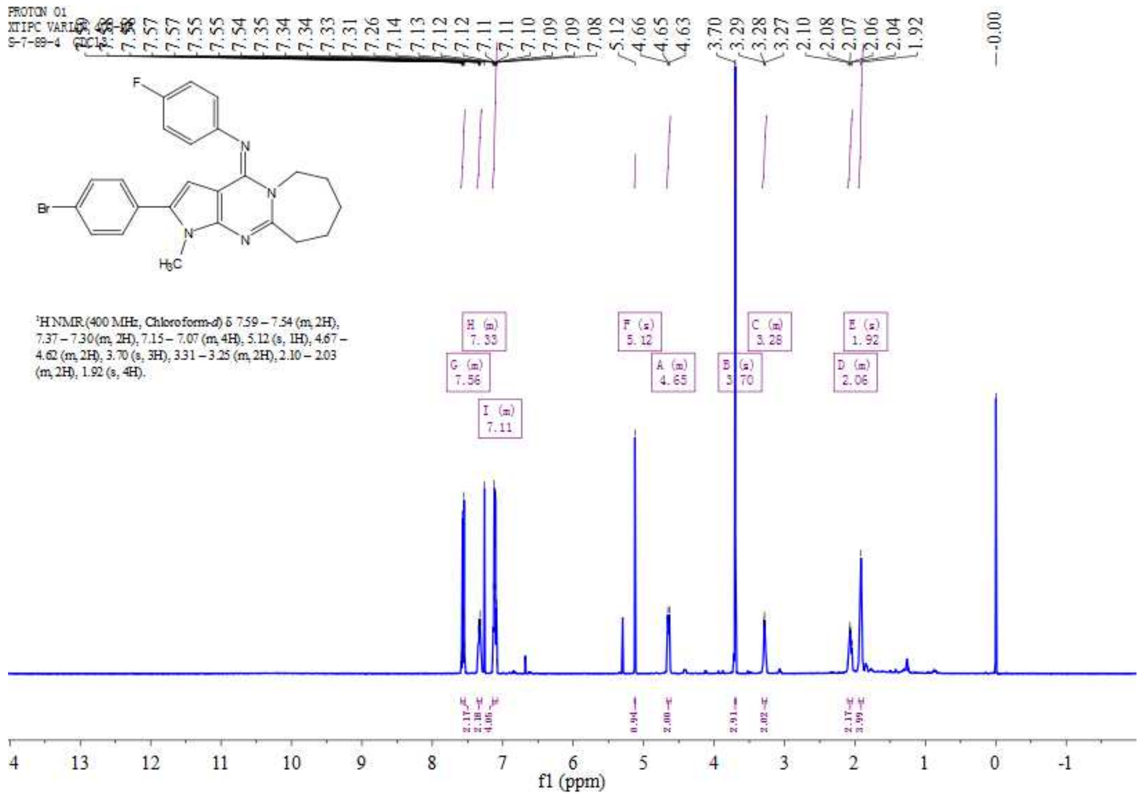

Figure S19. ^{1}H NMR spectrum of **8g**

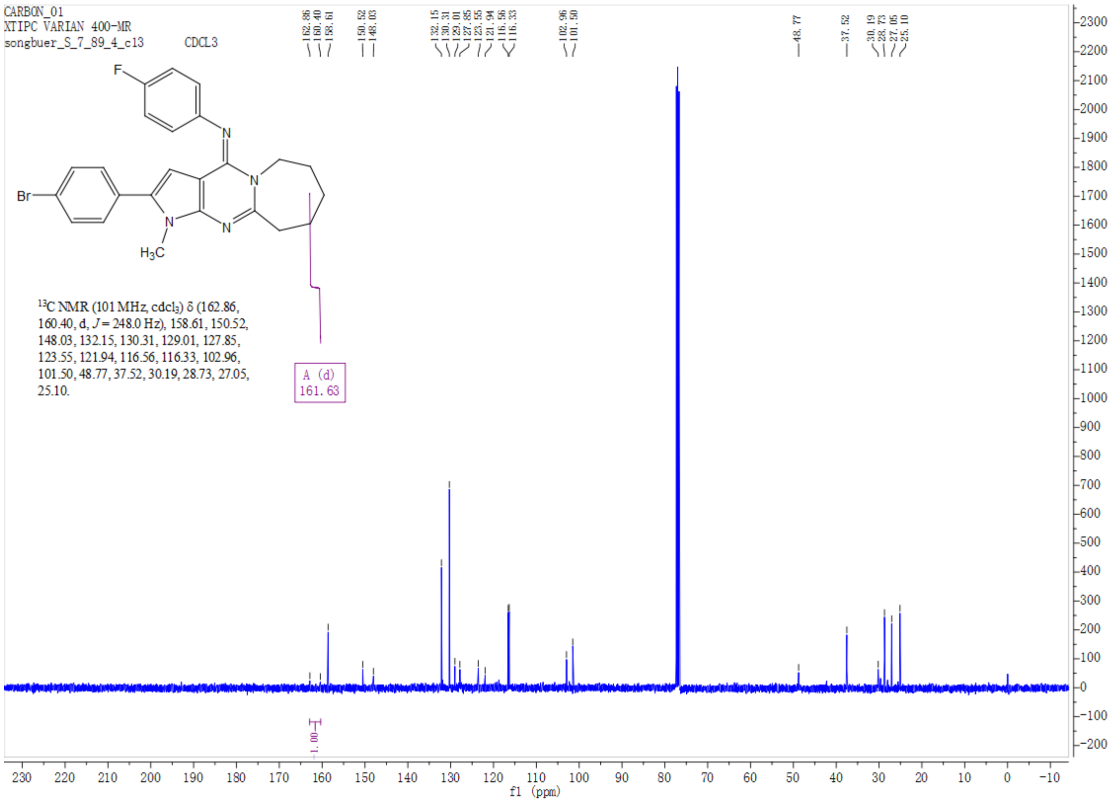

δ 162.86 and 160.40 are the coupling of fluorine atoms to carbon in a ratio of 1:1

Figure S20. ^{13}C NMR spectrum of **8g**

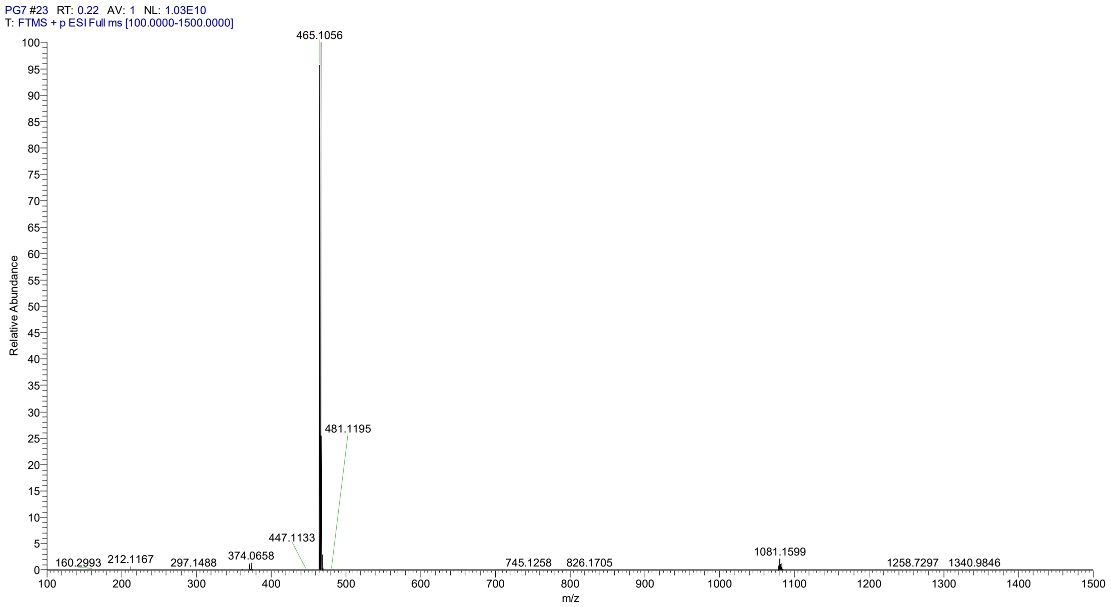

Figure S21. HRMS spectrum of **8g**

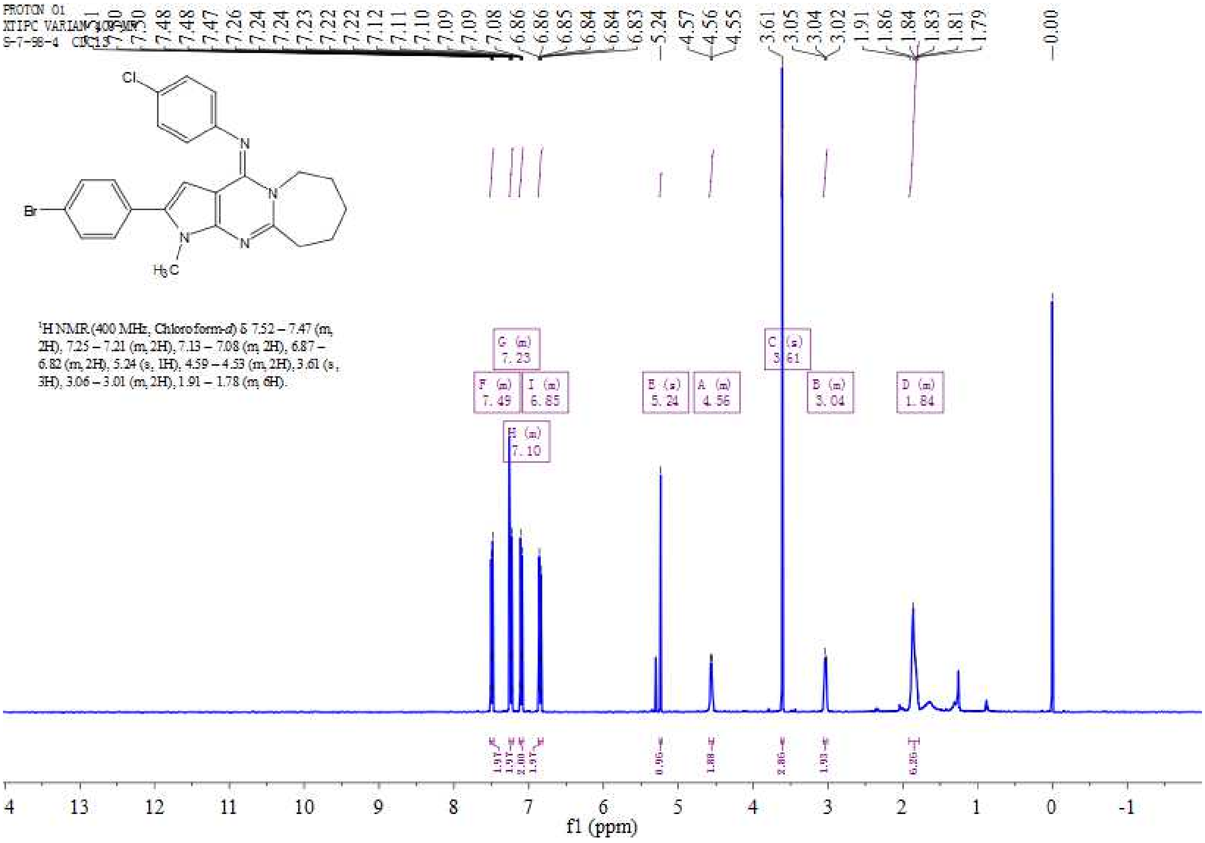

Figure S22. ^{1}H NMR spectrum of **8h**

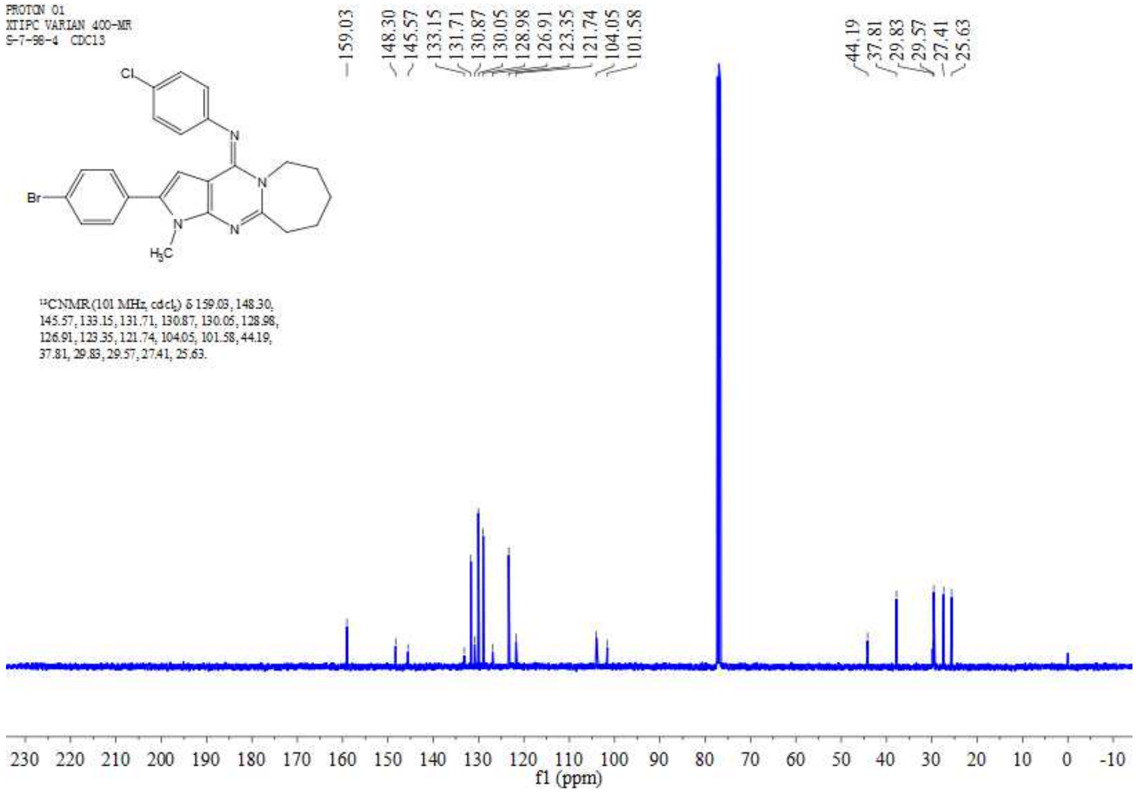

Figure S23. ^{13}C NMR spectrum of **8h**

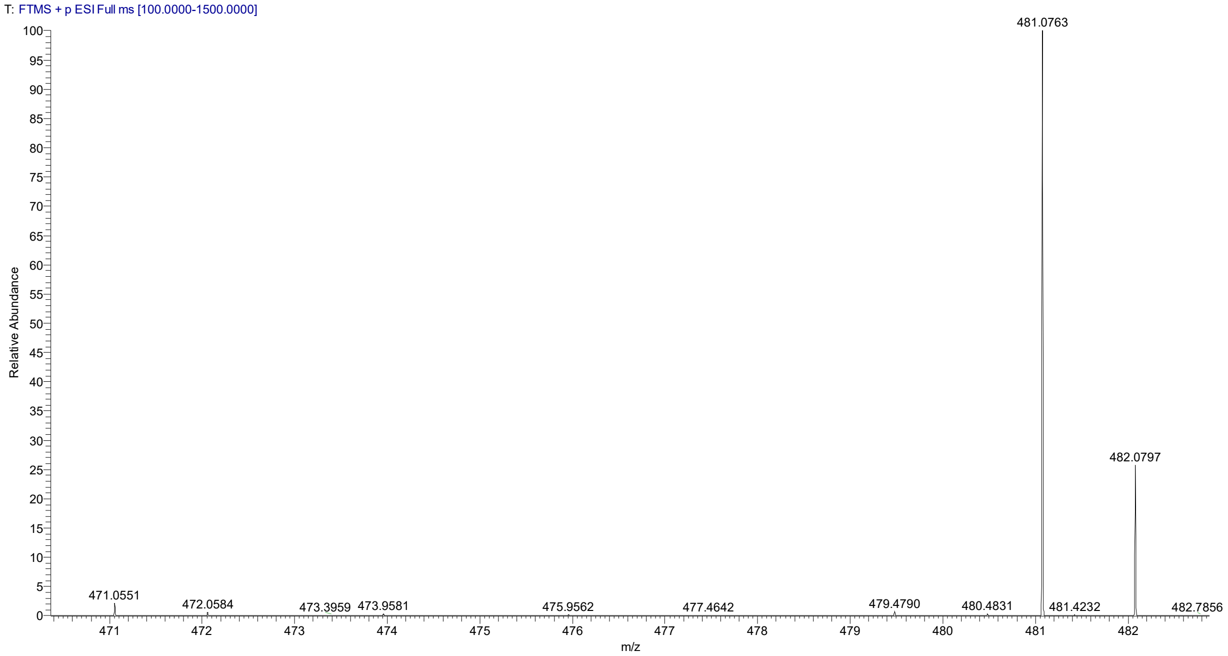

Figure S24. HRMS spectrum of **8h**

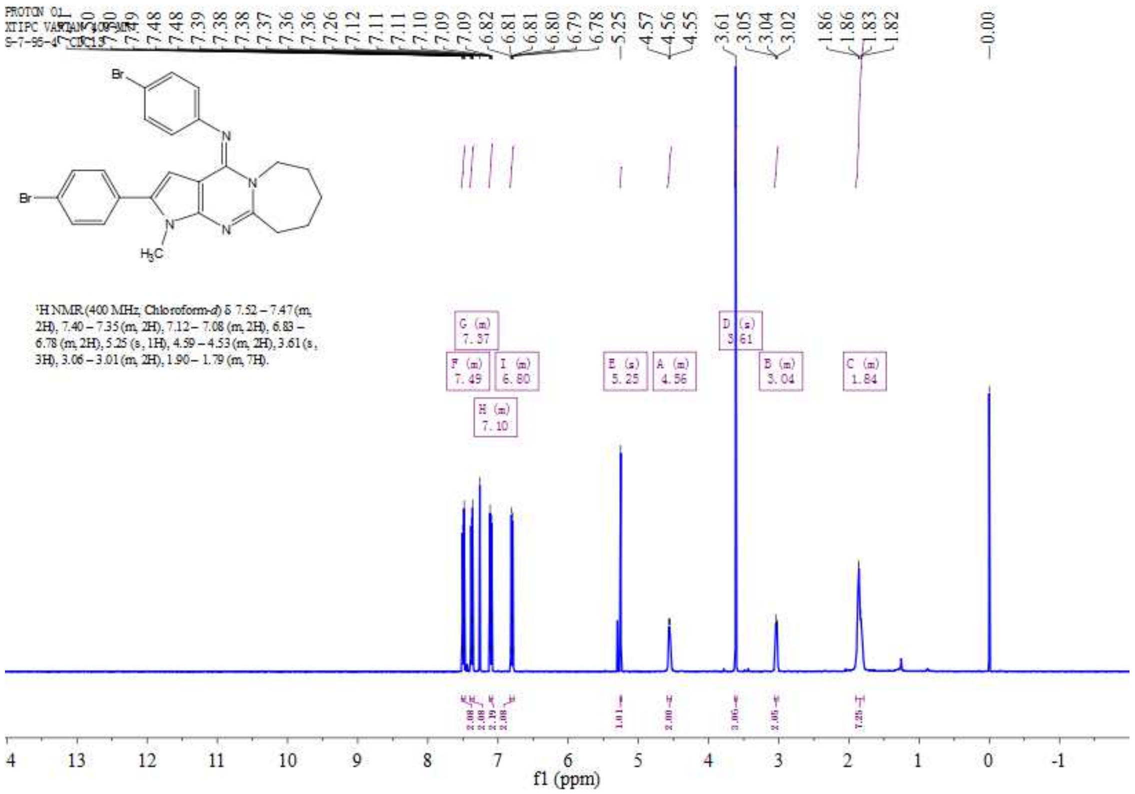

Figure S25. ^{1}H NMR spectrum of **8i**

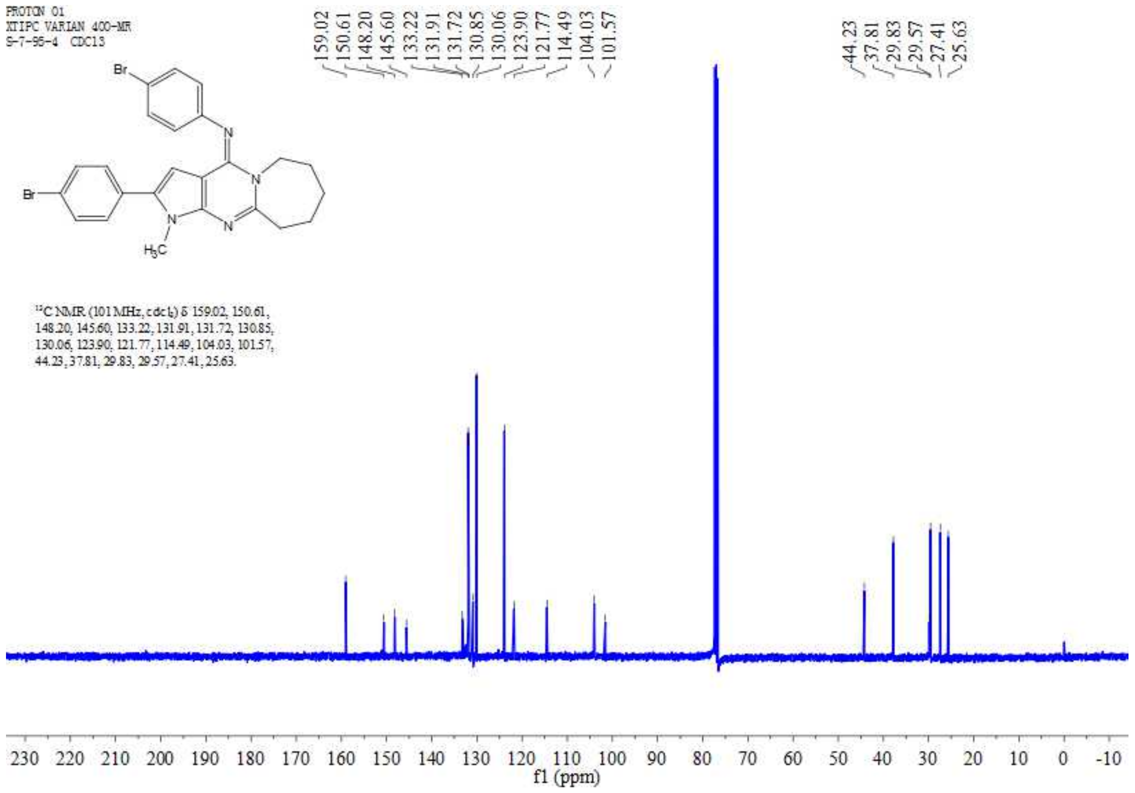

Figure S26. ^{13}C NMR spectrum of **8i**

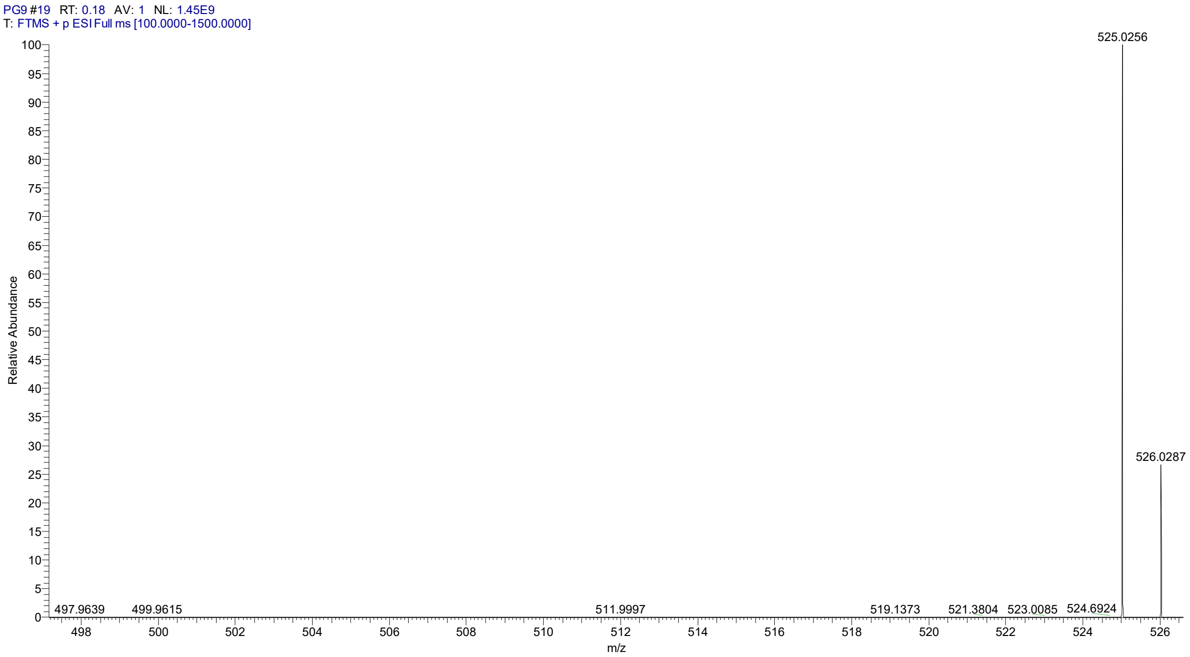

Figure S27. HRMS spectrum of **8i**

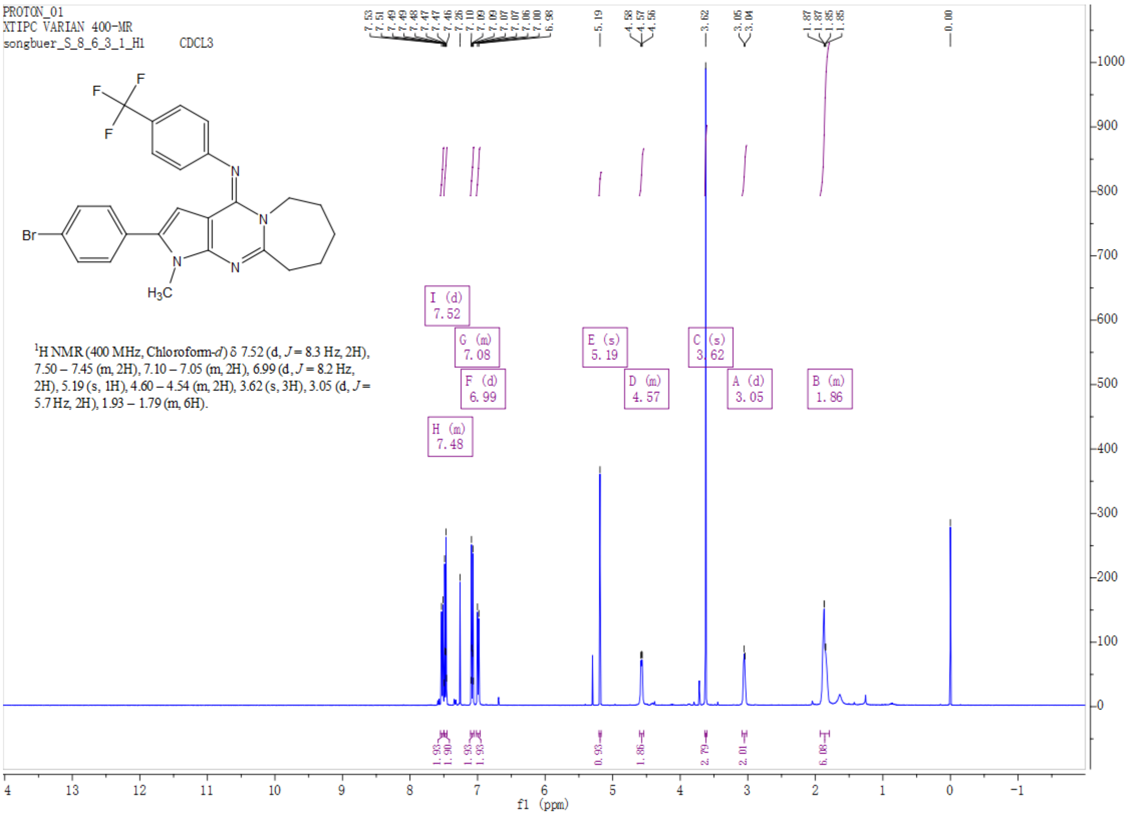

^{1}H NMR (400 MHz, Chloroform-*d*) δ 7.52 (d, *J* = 8.3 Hz, 2H), 7.50 – 7.45 (m, 2H), 7.10 – 7.05 (m, 2H), 6.99 (d, *J* = 8.2 Hz, 2H), 5.19 (s, 1H), 4.60 – 4.54 (m, 2H), 3.62 (s, 3H), 3.05 (d, *J* = 5.7 Hz, 2H), 1.93 – 1.79 (m, 6H).

Figure S28. ^{1}H NMR spectrum of **8j**

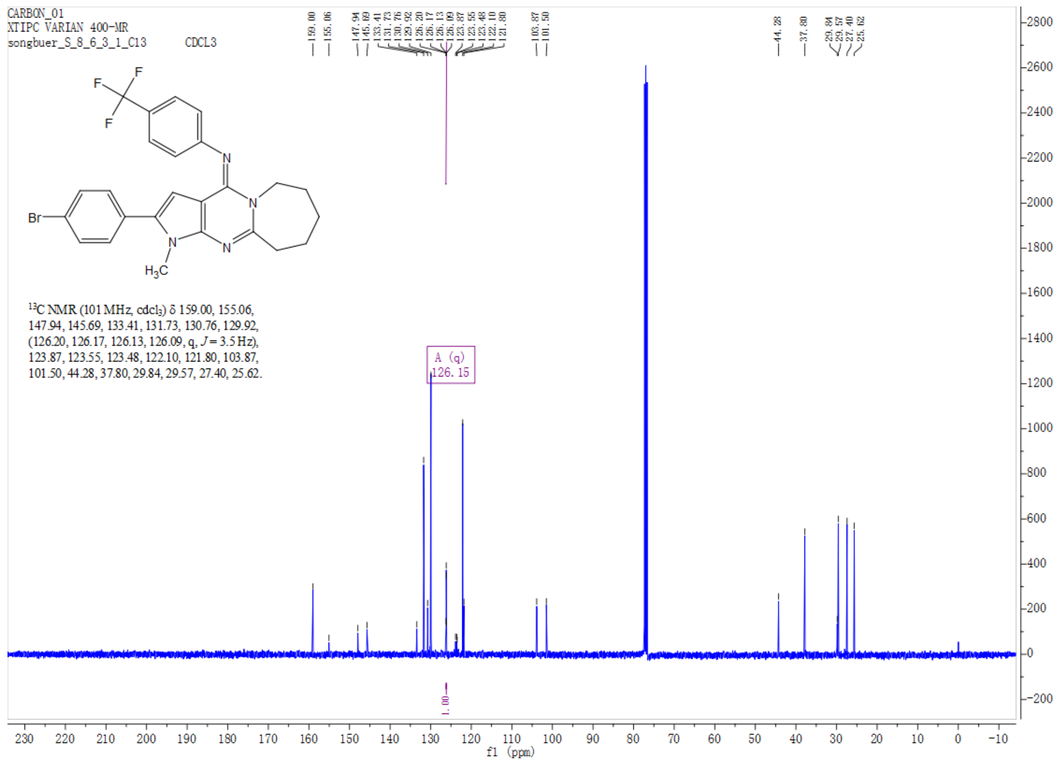

δ 126.20, 126.17, 126.13, 126.09, for the coupling splitting of trifluoromethyl to carbon, the ratio is 1:3:3:1

Figure S29. ^{13}C NMR spectrum of **8j**

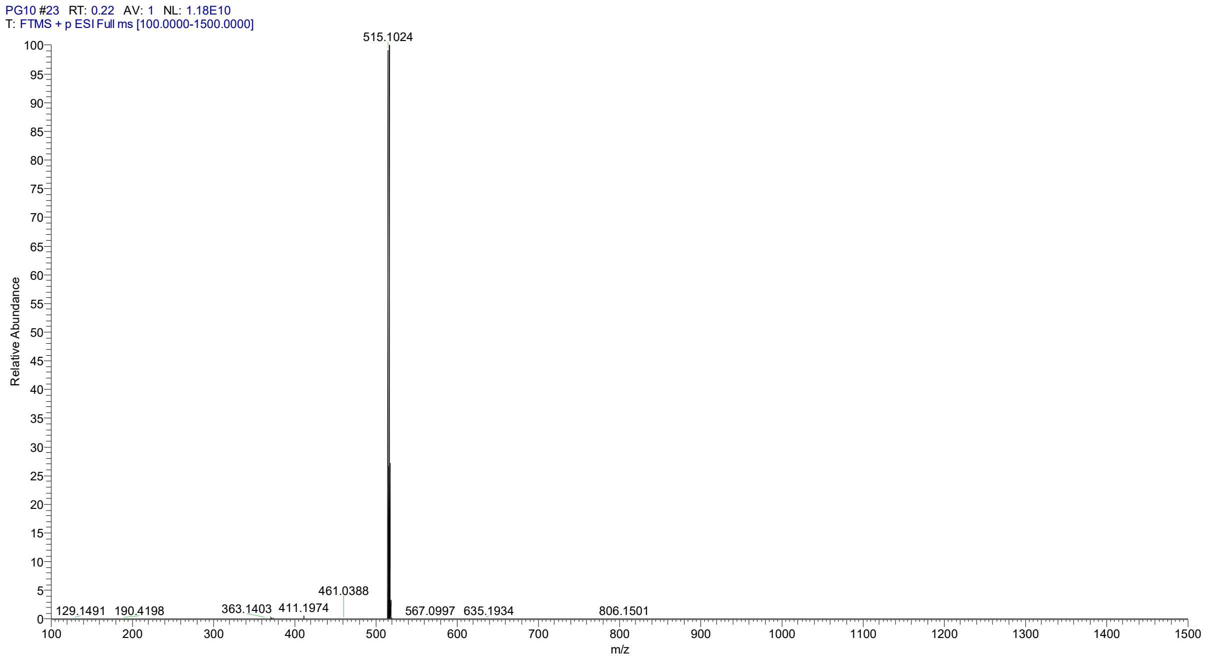

Figure S30. HRMS spectrum of **8j**

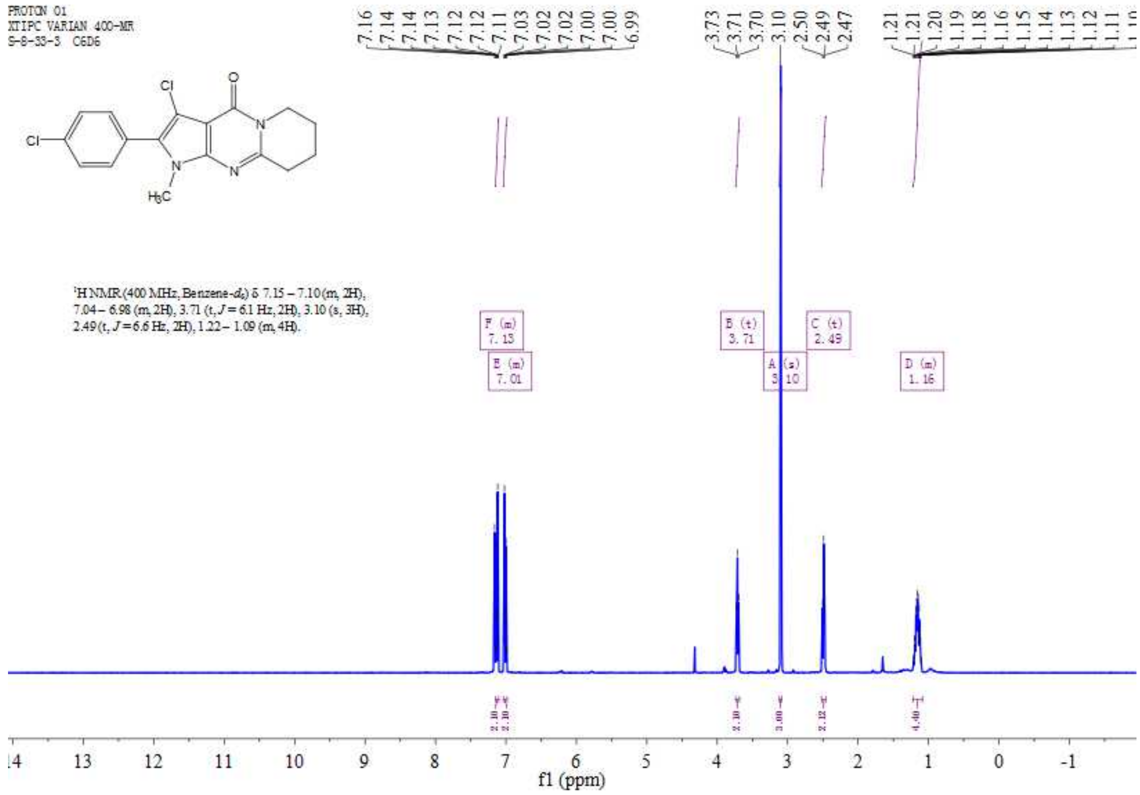

Figure S31. ^{1}H NMR spectrum of **10a**

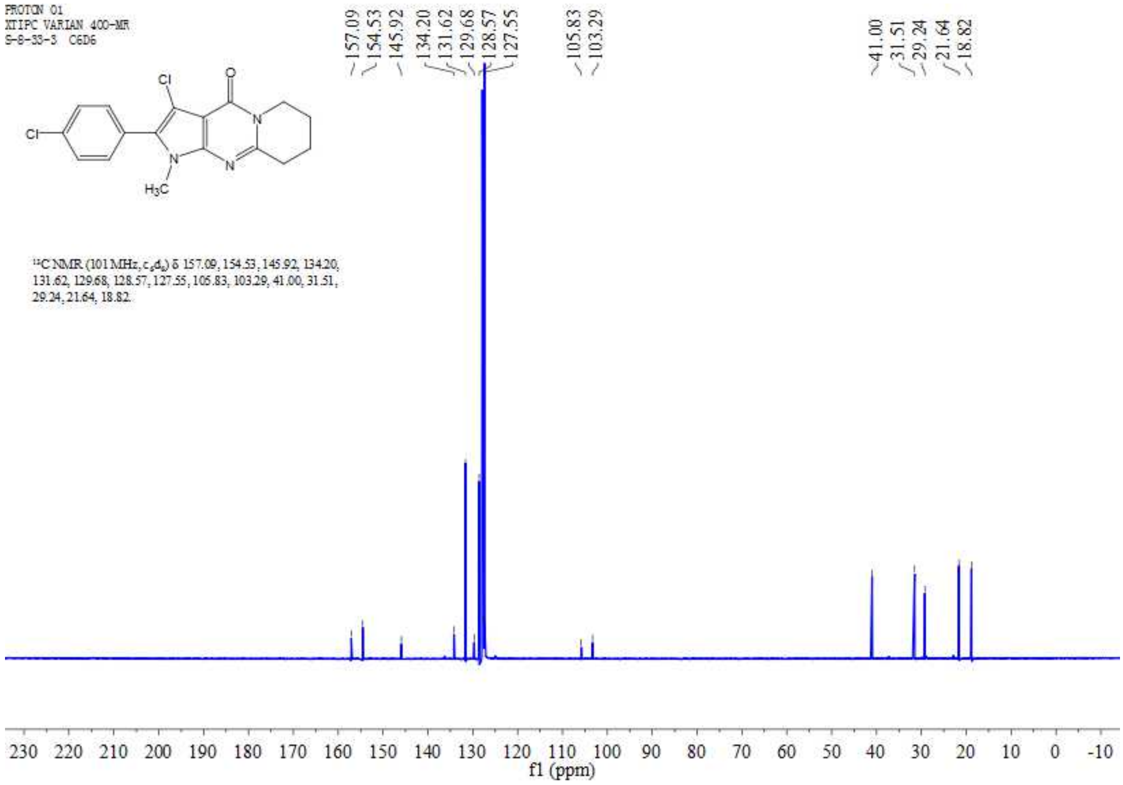

Figure S32. ^{13}C NMR spectrum of **10a**

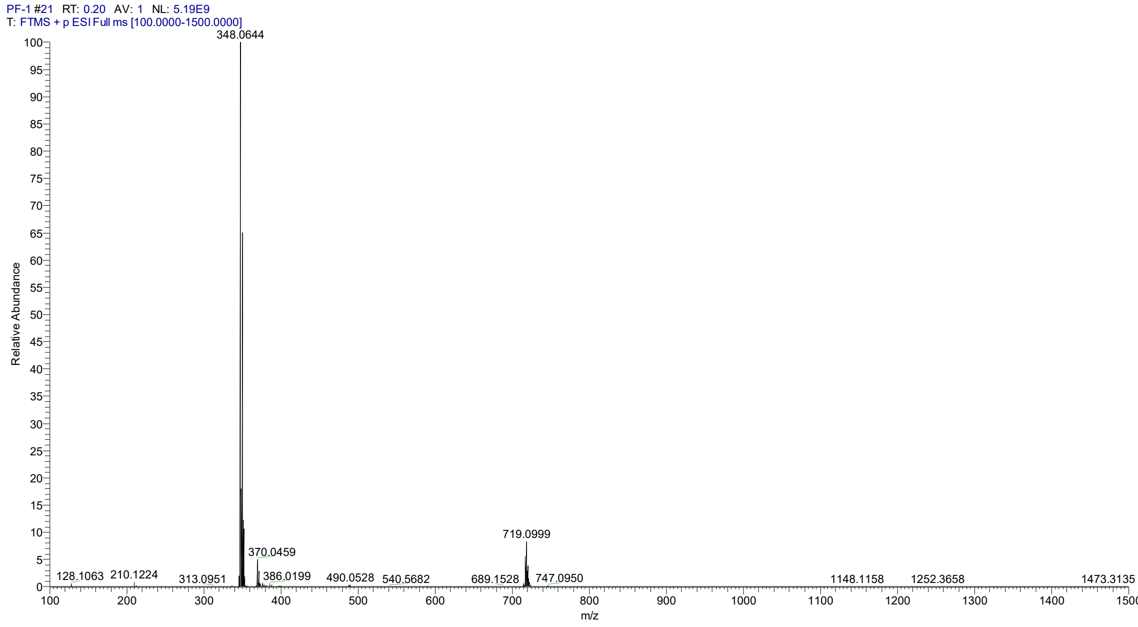

Figure S33. HRMS spectrum of **10a**

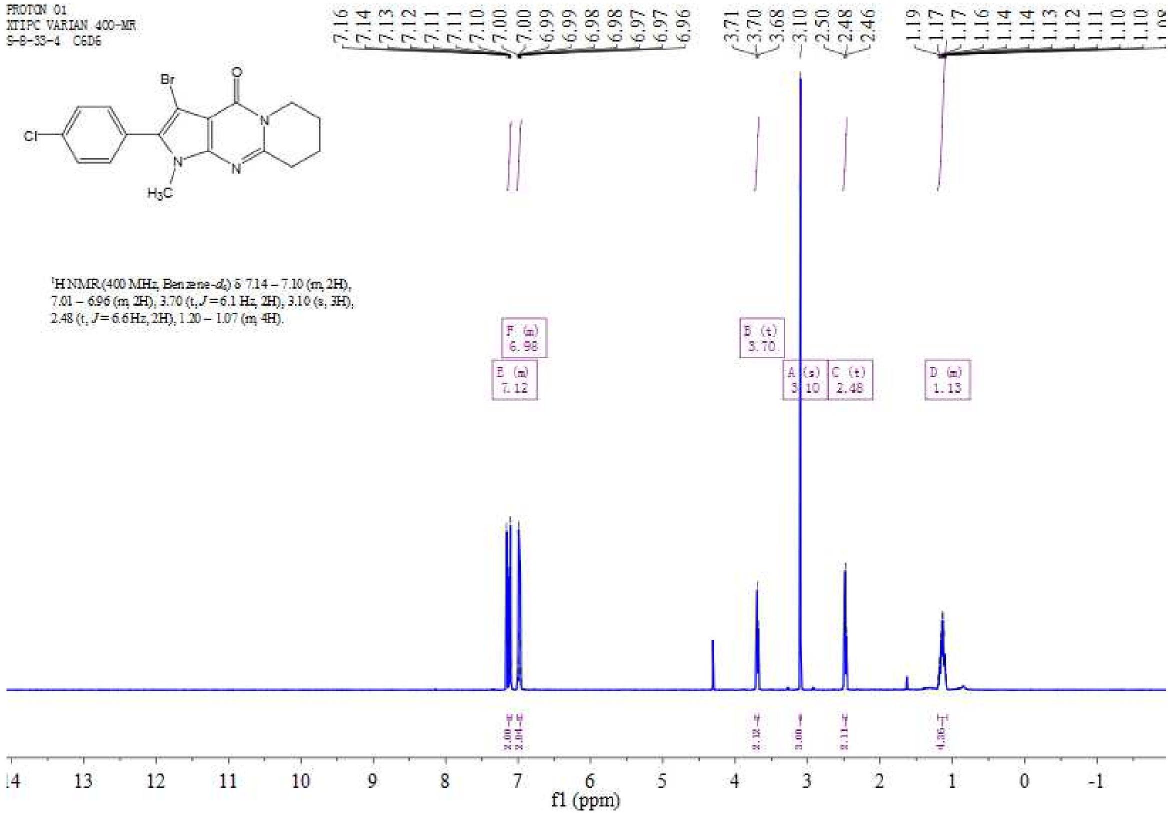

Figure S34. ¹H NMR spectrum of **10b**

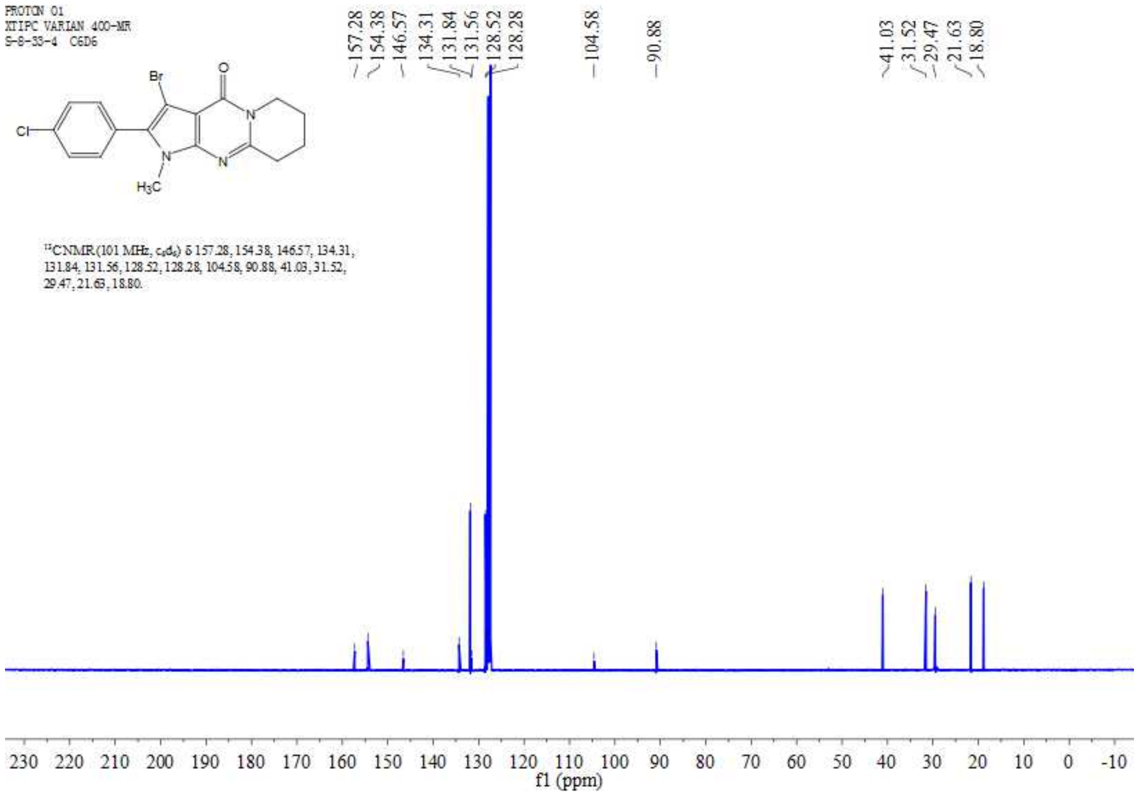

Figure S35. ^{13}C NMR spectrum of **10b**

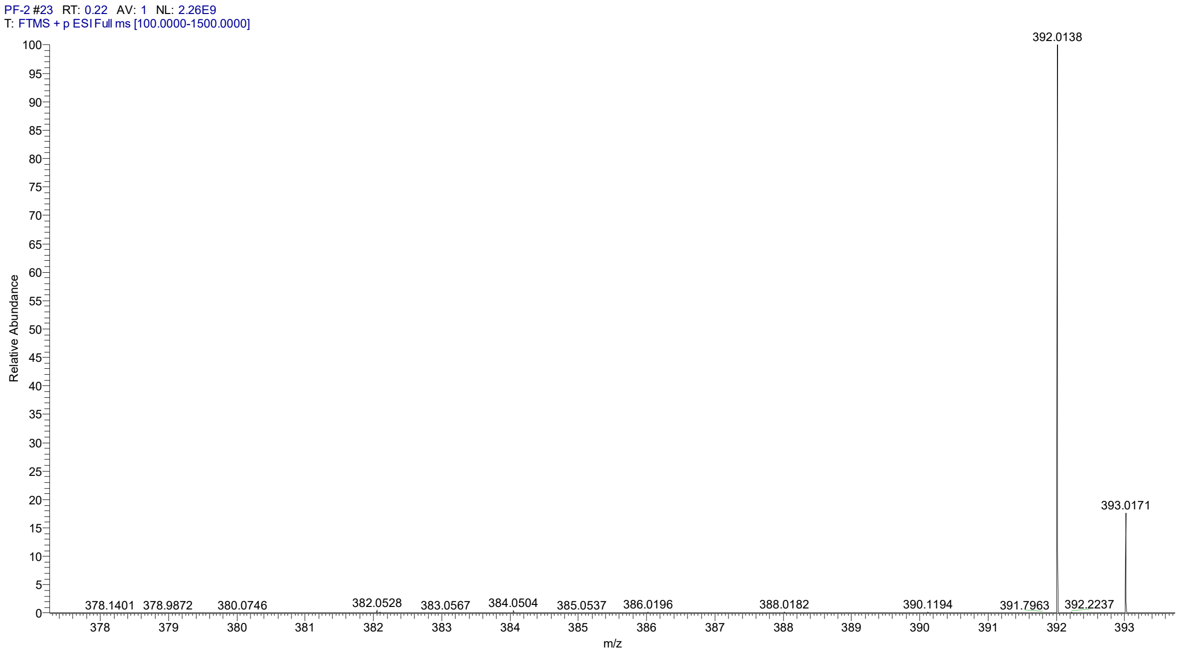

Figure S36. HRMS spectrum of **10b**

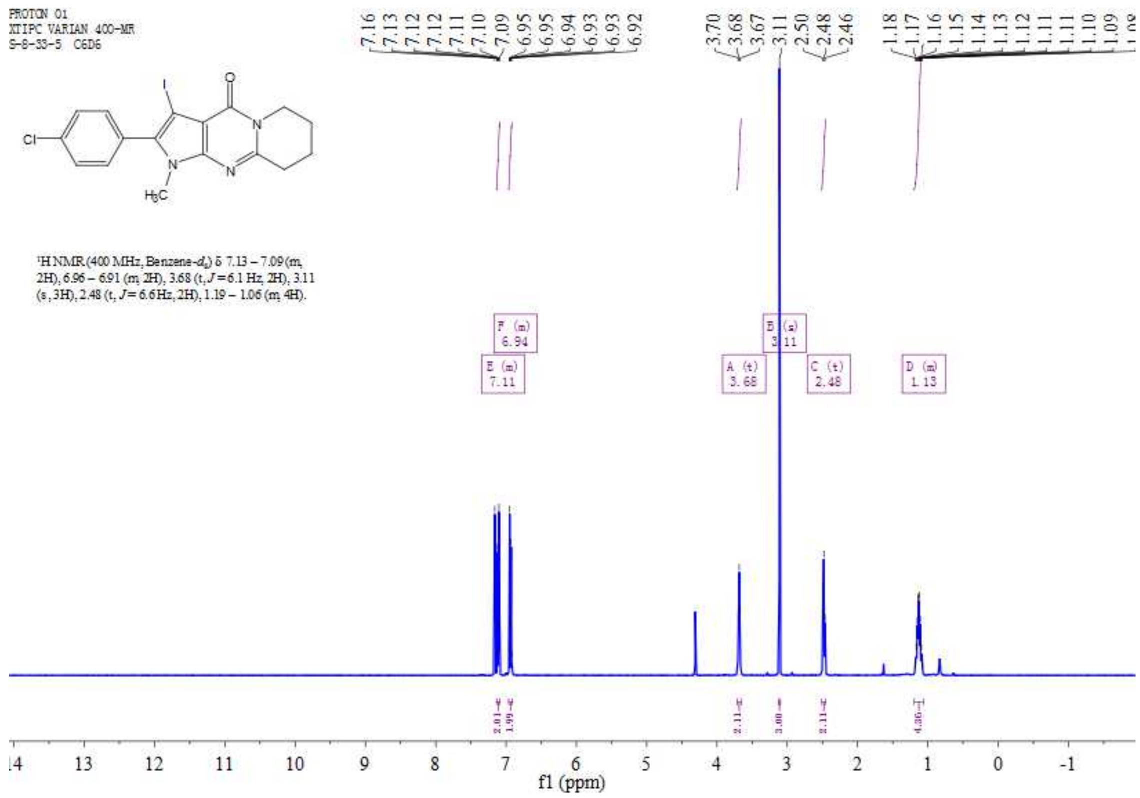

Figure S37. ^{1}H NMR spectrum of **10c**

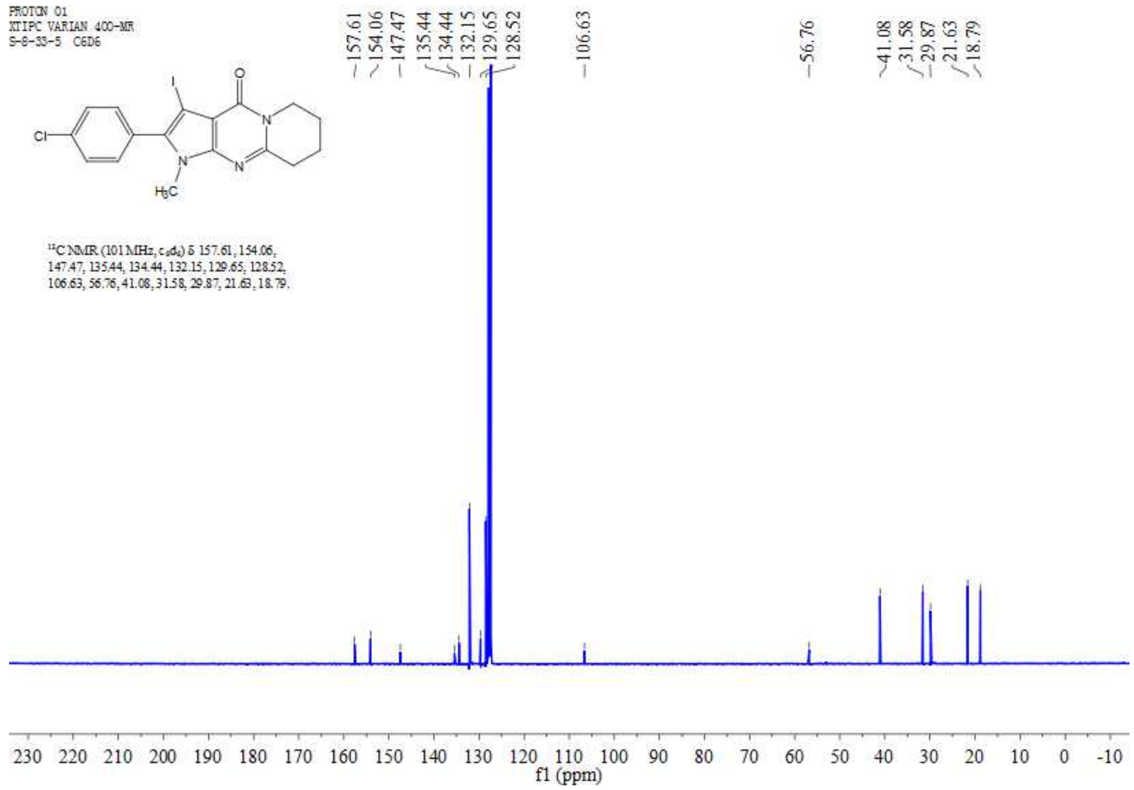

Figure S38. ^{13}C NMR spectrum of **10c**

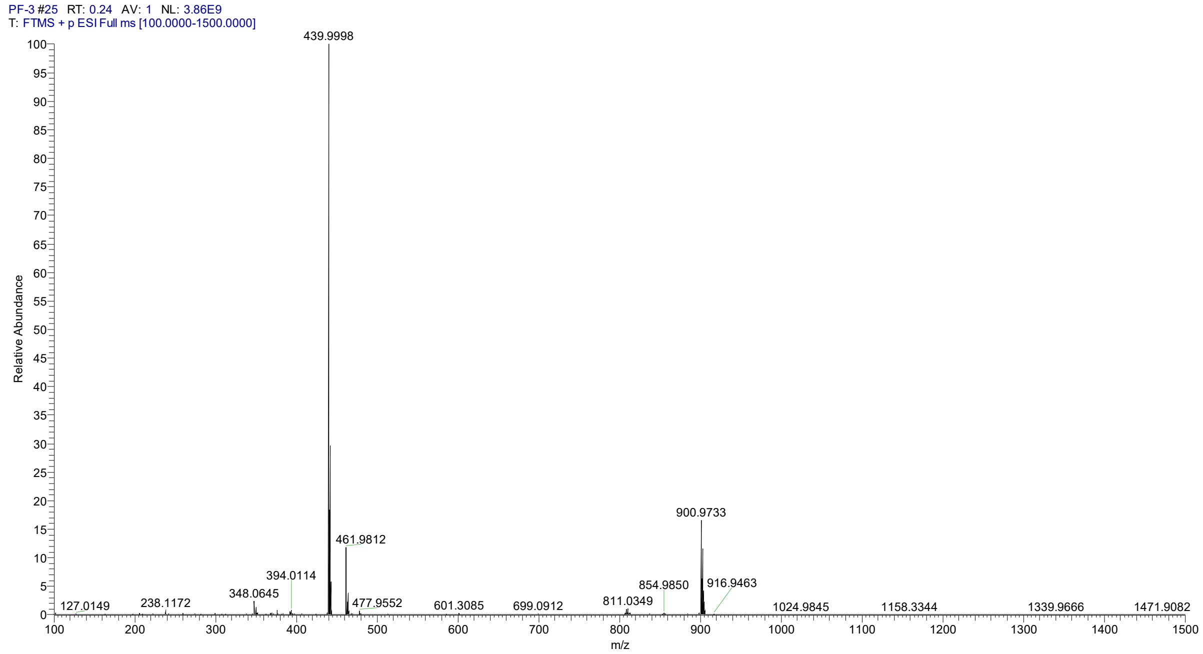

Figure S39. HRMS spectrum of **10c**

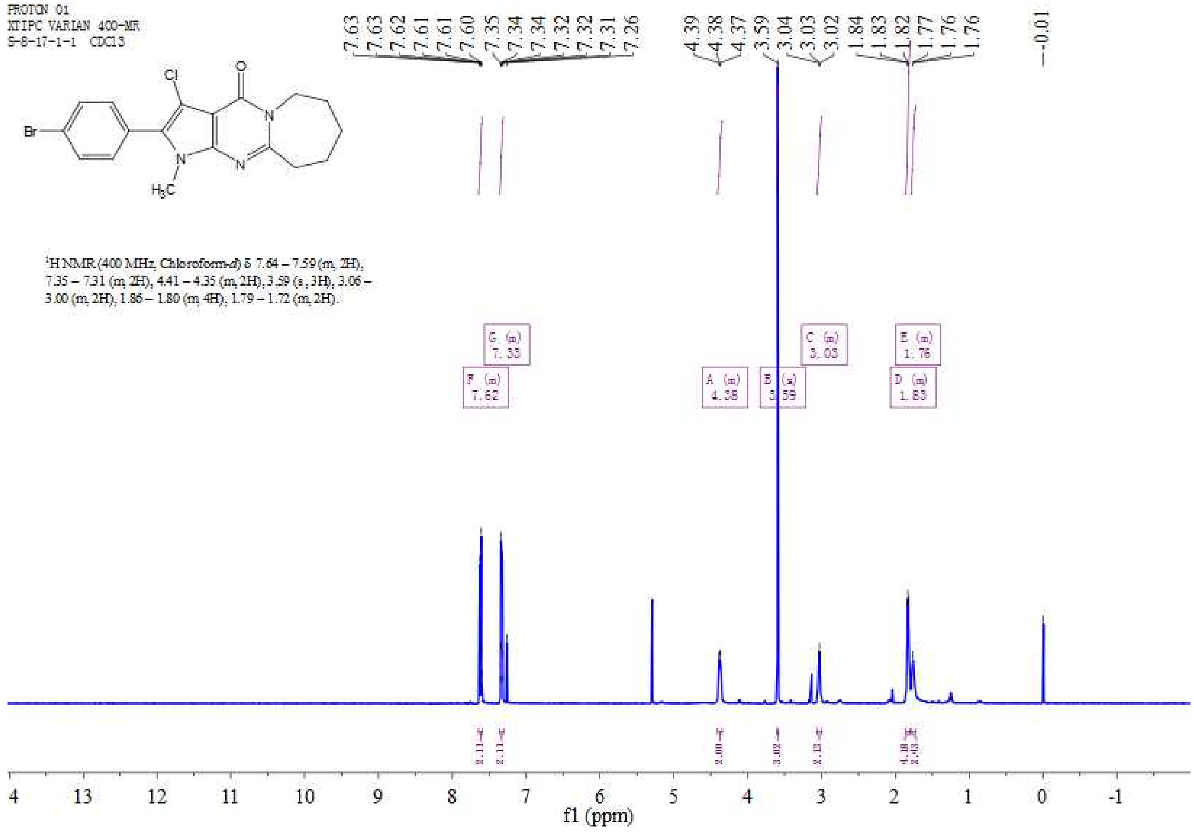

Figure S40. ^{1}H NMR spectrum of **10d**

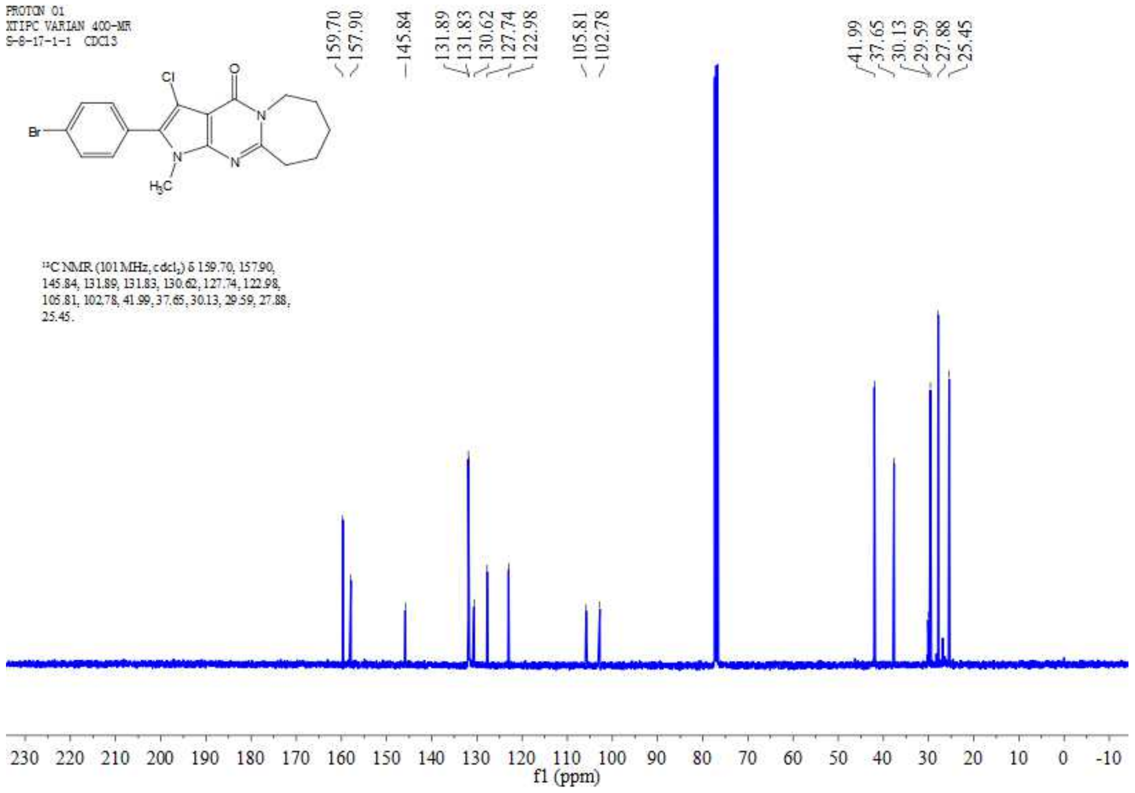

Figure S41. ^{13}C NMR spectrum of **10d**

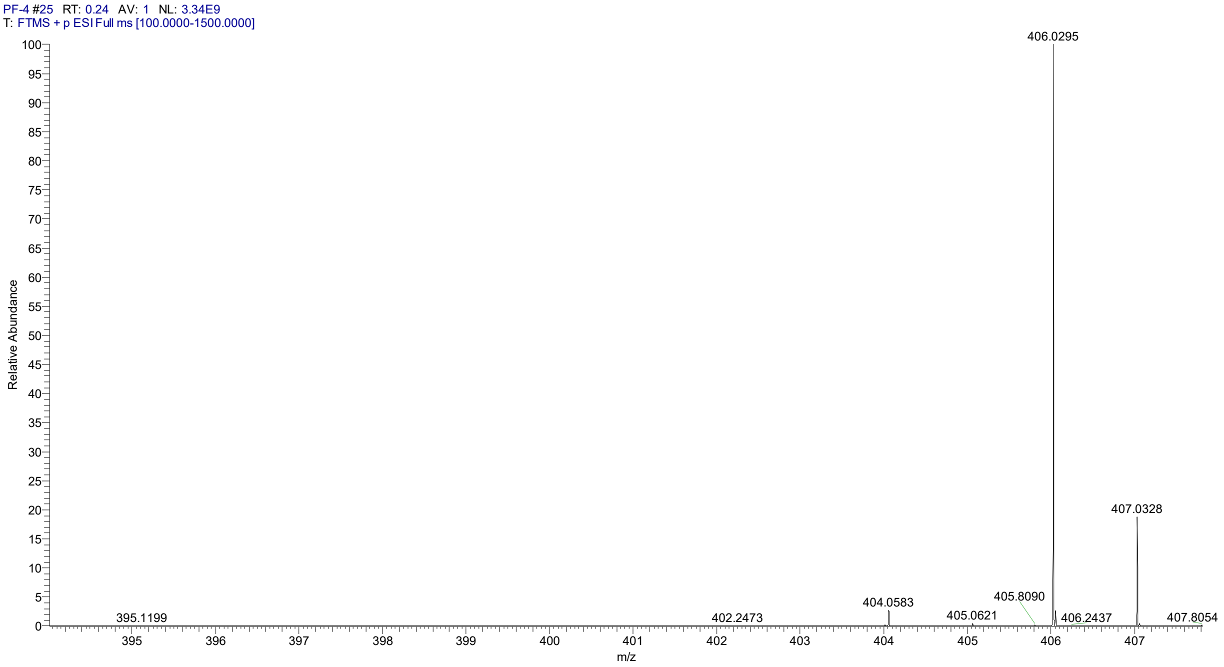

Figure S42. HRMS spectrum of **10d**

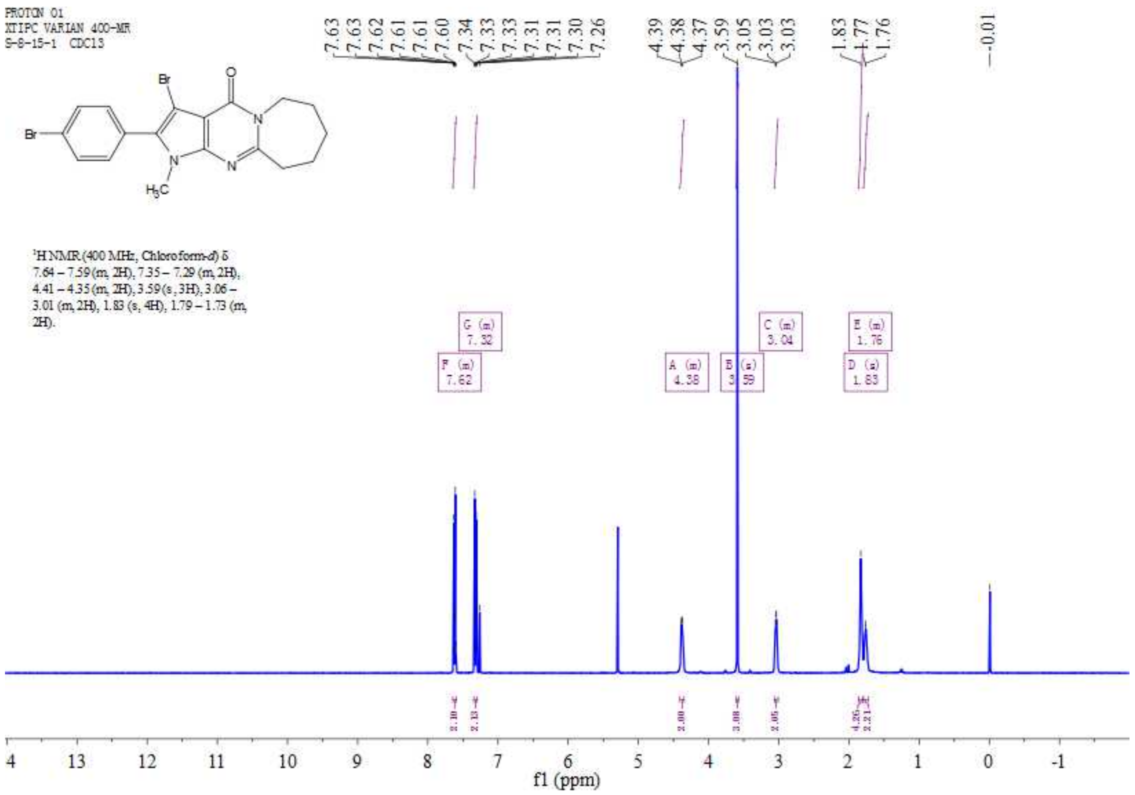

Figure S43. ^{1}H NMR spectrum of **10e**

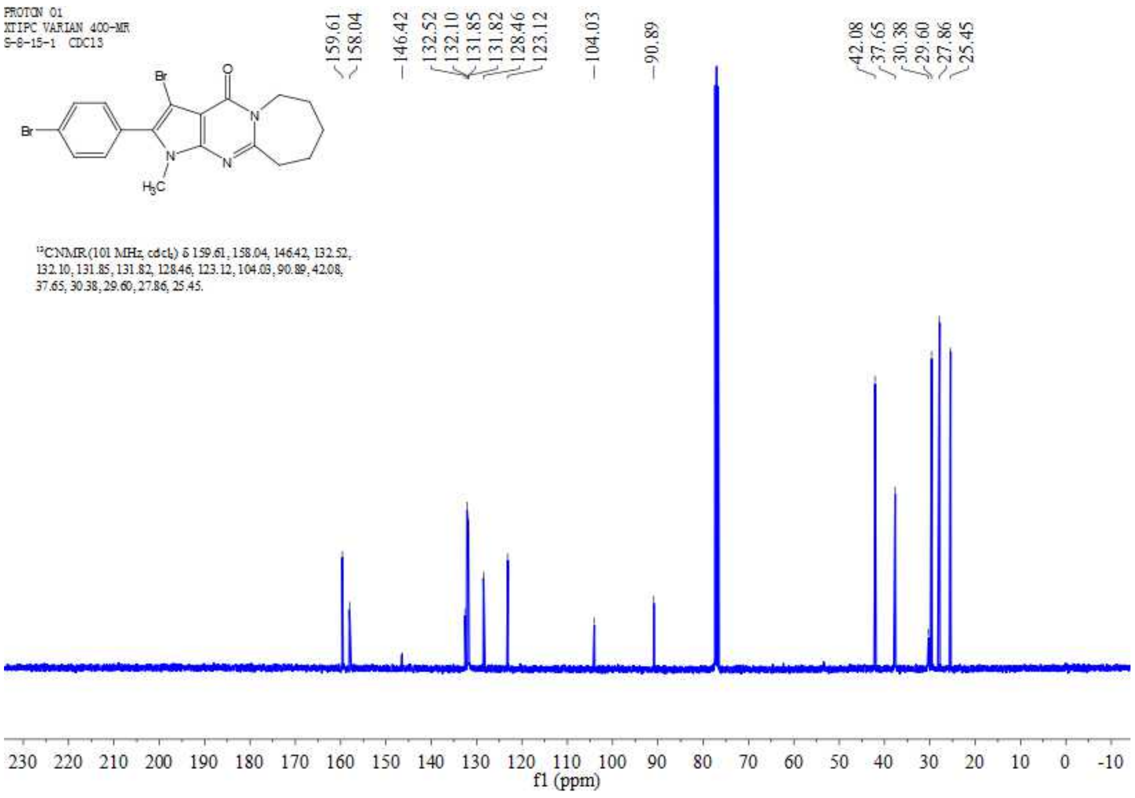

Figure S44. ^{13}C NMR spectrum of **10e**

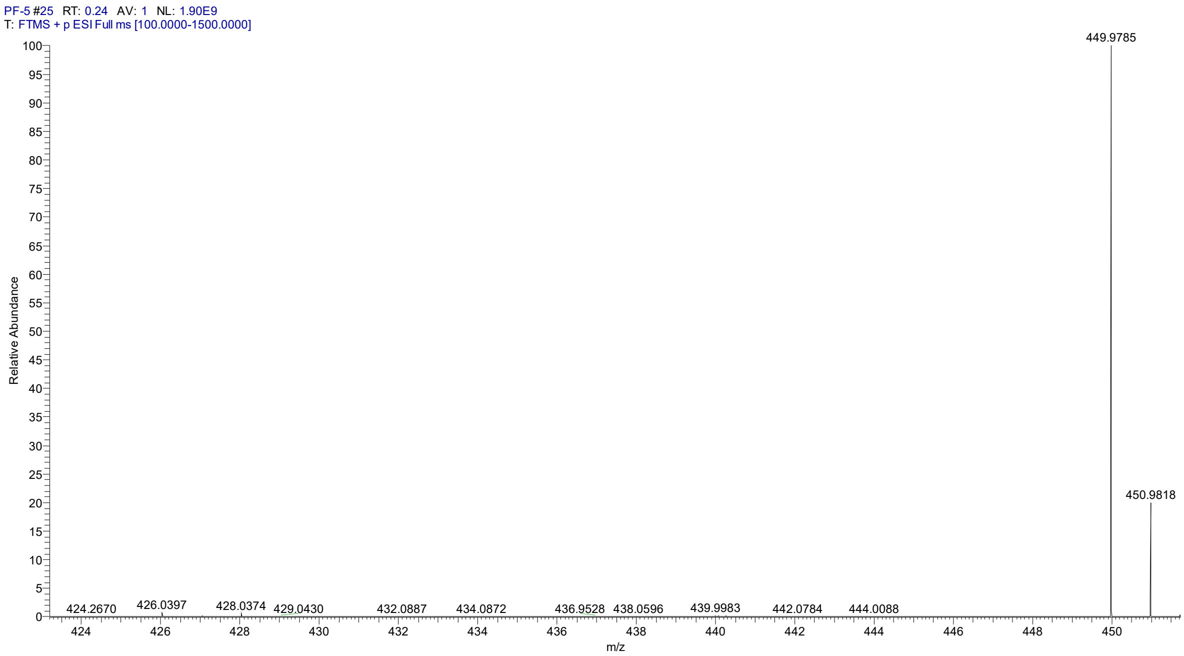

Figure S45. HRMS spectrum of **10e**

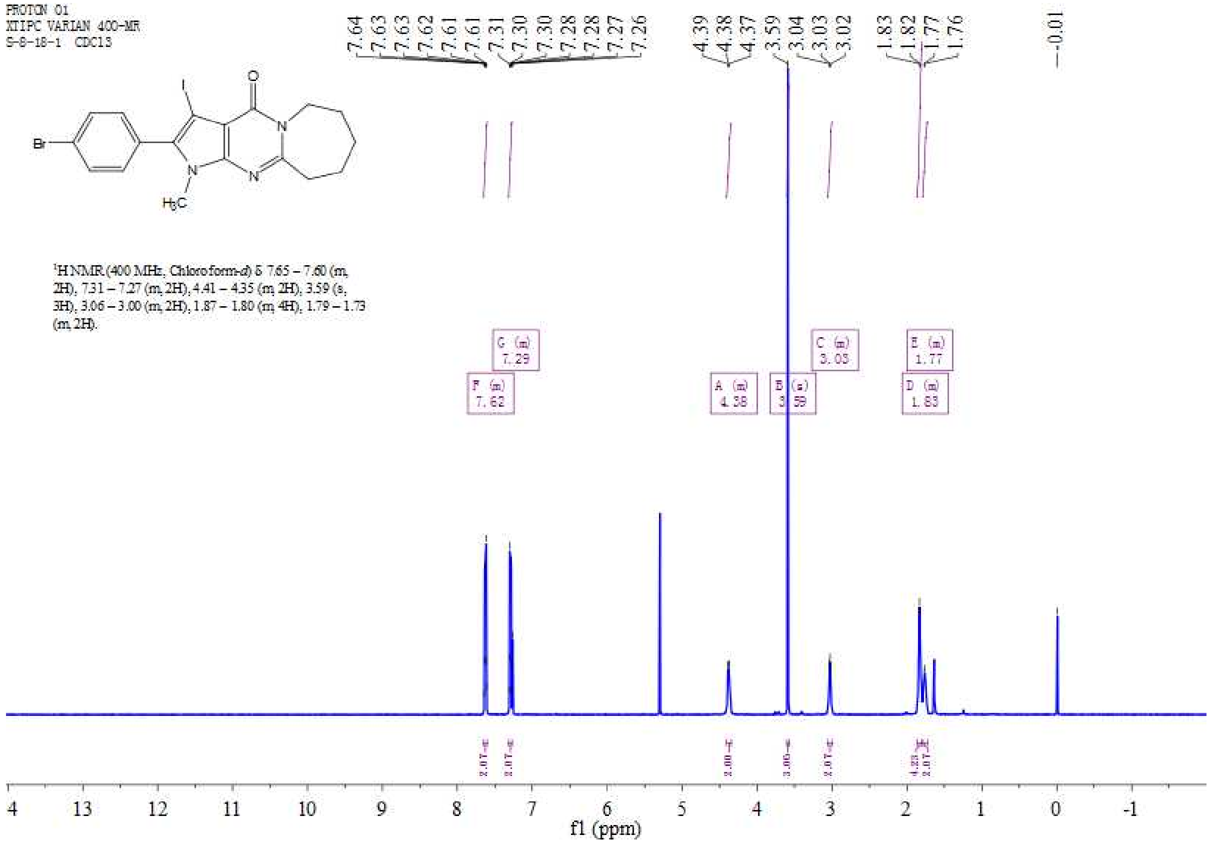

Figure S46. ^{1}H NMR spectrum of **10f**

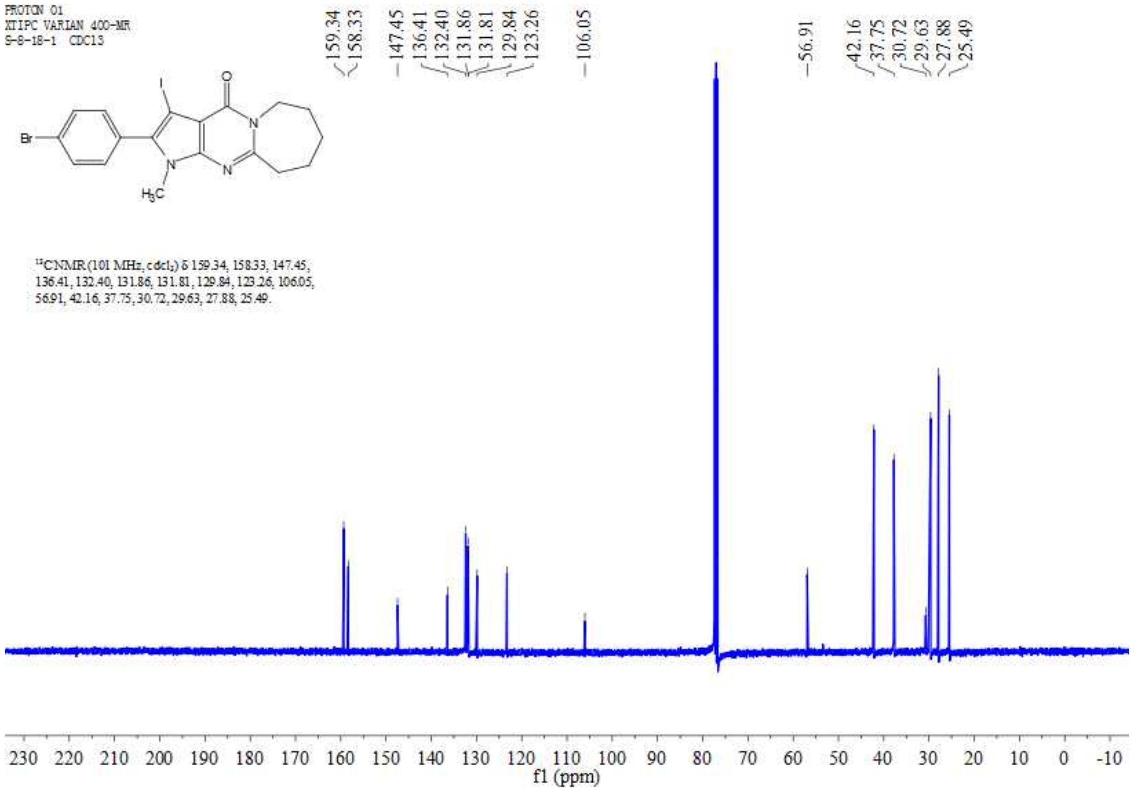

Figure S47. ^{13}C NMR spectrum of **10f**

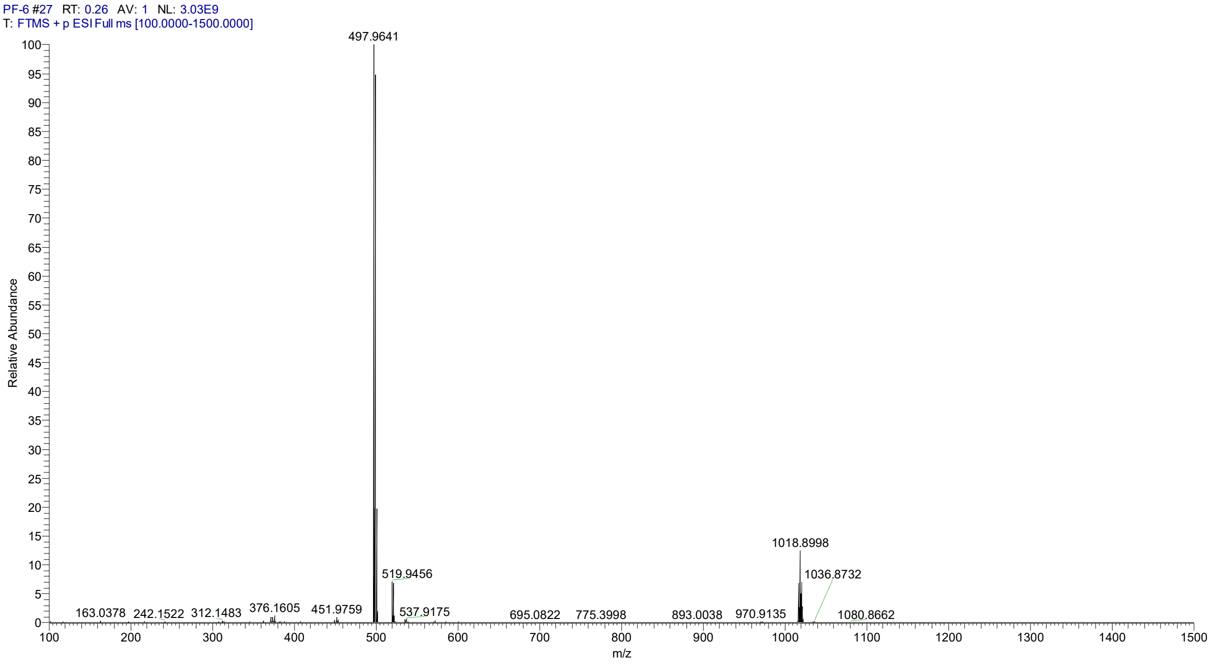

Figure S48. HRMS spectrum of **10f**
